# Selective clearance of aberrant membrane proteins by TORC1-mediated micro-ER-phagy

**DOI:** 10.1016/j.celrep.2025.115282

**Published:** 2025-02-12

**Authors:** Valeriya Gyurkovska, Yaneris M. Alvarado Cartagena, Rakhilya Murtazina, Sarah F. Zhao, Candela Ximenez de Olaso, Nava Segev

**Affiliations:** 1 Department of Biochemistry and Molecular Genetics, College of Medicine, University of Illinois at Chicago, Chicago, IL, USA; 2 These authors contributed equally; 3 Lead contact

## Abstract

Aberrant accumulation and clearance of membrane proteins is associated with disease. Membrane proteins are inserted first to the endoplasmic reticulum (ER). During normal growth, two quality control (QC) processes, ER-associated degradation and macro-ER-phagy, deliver misfolded and excess membrane proteins for degradation in the proteasome and lysosome, respectively. We show that in yeast during normal growth, ER-QC is constitutive, since none of the stress-induced signaling pathways—nutritional, proteotoxic, or heat—are involved. In mutant cells defective in ER-QC, misfolded or excess proteins accumulate and nutritional stress, but not proteotoxic or heat stress, can stimulate their clearance. Early during nutritional stress, clearance occurs in the lysosome through a selective micro-ER-phagy pathway dependent on the ubiquitin ligase Rsp5, its Ssh4 adaptor, and ESCRT. In contrast, only a fraction of normal membrane proteins is degraded much later via macro-autophagy. Because the pathways explored here are conserved, nutritional stress emerges as a possible way for clearing disease-associated membrane proteins.

## In brief

Gyurkovska et al. identify a micro-ER-phagy pathway that clears aberrant, but not normal, membrane proteins under nutritional stress and provide a live glimpse of engulfment of cargo by the lysosome. This pathway occurs before degradation of normal proteins by macroautophagy and requires a ubiquitin ligase and the ESCRT complex.

## Graphical abstract

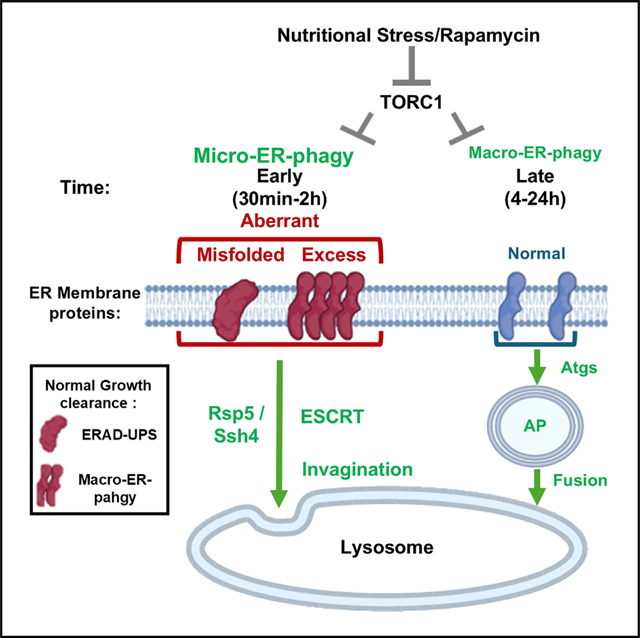

## INTRODUCTION

Membrane-embedded proteins comprise about one-third of the human proteome, and their excess or mutations that affect their folding result in human disease.^1,2^ Most of these proteins are inserted first into the endoplasmic reticulum (ER), where during normal growth, two different quality control (QC) pathways recycle misfolded and excess proteins from the ER by delivering them to one of the two cellular degradative compartments.^3^ ER-associated degradation (ERAD) delivers cargo to the proteasome,^4,5^ while the macro-ER-autophagy pathway, macro-ER-phagy, delivers its cargo to the lysosome (vacuole in yeast).^6^ In the proteasome and lysosome, degradative enzymes break down macromolecular cargoes to their building blocks for their reuse. We consider the lysosome as the top of the cellular predatory chain because, whereas the proteasome can only degrade proteins, the lysosome can degrade all types of macromolecules and cellular compartments, including proteasomes.^7^

In the ERAD pathways, proteins containing a degron, a domain necessary and sufficient to target a stable protein for degradation by the ubiquitin-proteasome system (UPS), are ubiquitinated by E3 ligases and degraded by the UPS.^8^ Alternatively, all types of macromolecules, including proteasomes and cellular organelles, can be degraded in the lysosome,^9^ especially under stress. Macro-ER-phagy is a selective macro-autophagy pathway, which requires specific receptors, is mediated by the conserved ATG proteins and autophagosomes (APs), and delivers ER fragments for degradation in the lysosome.^10,11^ In contrast, in micro-autophagy cargo reaches the lysosome via direct engulfment by the lysosomal membrane and is independent of core autophagy-related proteins (Atgs) and APs.^12^ Currently very little is known about micro-autophagy regulation and mechanisms.^13^

Under normal growth conditions, when an individual membrane protein contains a degron or is overexpressed, cells need to clear it. Yeast cells do this efficiently while continuing their normal growth, and the only way to know that this has occurred is to block the relevant pathway by mutations or drugs. For example, an individual degron-containing protein accumulates in cells defective in ERAD,^14^ and an individual overex-pressed membrane protein accumulates in the ER of cells defective in macro-autophagy.^6,15^ ERAD-C and macro-ER-phagy can help clear each other’s cargoes.^15^ One remaining question is whether clearance of individual membrane proteins under normal growth conditions is constitutive or whether stress-induced signaling pathways that can stimulate ERAD and/or autophagy play a role in such clearance.

Cellular stresses, nutritional and proteotoxic (or ER), stimulate the signaling cascades target-of-rapamycin complex 1 (TORC1) and the unfolded protein response (UPR), respectively. These signaling pathways are important for ramping up autophagy, including macro-ER-phagy and ERAD, respectively, to deal with heavy loads of multiple cargoes that need to be cleared under stress.^16,17^ As for micro-ER-phagy, it was studied mostly in the context of proteotoxic stress.^12,18^ Therefore, a second question is whether nutritional and ER stresses, and their corresponding signaling pathways TORC1 and UPR, can help clearance of each other’s cargo. Another type of cellular stress, heat, causes accumulation of massive amounts of misfolded and aggregated membranes, which in turn induce a third protein QC signaling pathway, the heat-shock response (HSR).^19^ HSR increases the expression of protein chaperones that facilitate disaggregation of misfolded proteins, which are either refolded or degraded by the UPS pathway. In addition, under chronic stress conditions, aggrephagy, a selective autophagy pathway, can deliver misfolded protein aggregates for degradation in the lysosome.^20^ In addition to heat, other environmental stresses, such as ER stress, can also activate HSR and HSR, in turn, can relieve ER stress.^21^ Thus, the three QC pathways stimulated by the different stresses heat, ER-stress, and starvation can partially overlap to protect cells from proteotoxicity. Therefore, a third question addressed here is whether HSR is induced by excess (ER-phagy cargo) or misfolded (ERAD cargo) single-membrane ER proteins and whether heat and/or HSR can help clear these cargoes.

Here, we tested two types of aberrant membrane proteins, misfolded and excess. The degron-containing Deg1-Vma12-GFP is ubiquitinated by the ERAD Doa10 E3-ubiquitin ligase and degraded by the UPS during normal growth^14^ but accumulates in *doa10Δ* mutant cells. It serves as a model for misfolded proteins in which degradation elements are exposed and recognized by ERAD.^22^ During normal growth, overexpressed GFP-Snc1-PEM and Snq2-GFP, a single-path chimeric protein and a multi-path transporter, respectively, are degraded by macro-ER-phagy in the lysosome but accumulate in cells defective in macro-autophagy^6^ and serve as a model for excess membrane proteins. These were compared to Rtn1-GFP expressed endogenously, a substrate of macro-ER-phagy under rapamycin treatment,^23^ and to the mitochondrial marker Cox4-mCherry. Clearance was tested under different kinds of cellular stresses. We show that clearance of aberrant membrane proteins during normal growth is constitutive. Moreover, if aberrant membrane proteins accumulate, they can be cleared only under nutritional stress, but not ER or heat stress, through a selective micro-ER-phagy pathway, whereas a fraction of normal membrane proteins is degraded much later via macro-autophagy. The mechanism and dynamics of the selective micro-ER-phagy pathway were characterized. Because the recycling and signaling pathways are conserved from yeast to human cells,^24,25^ their functioning under different kinds of stress is relevant for the well-being of all cells, especially for the long-living neuronal cells.^26^ Our results point to the use of nutritional stress as a strategy for selective clearance of toxic membrane proteins from cells and highlight a novel micro-ER-phagy pathway for such clearance.

## RESULTS

### Micro-ER-phagy under nutritional stress

#### Clearance of an ERAD cargo during nutritional stress

**Figure 1.**
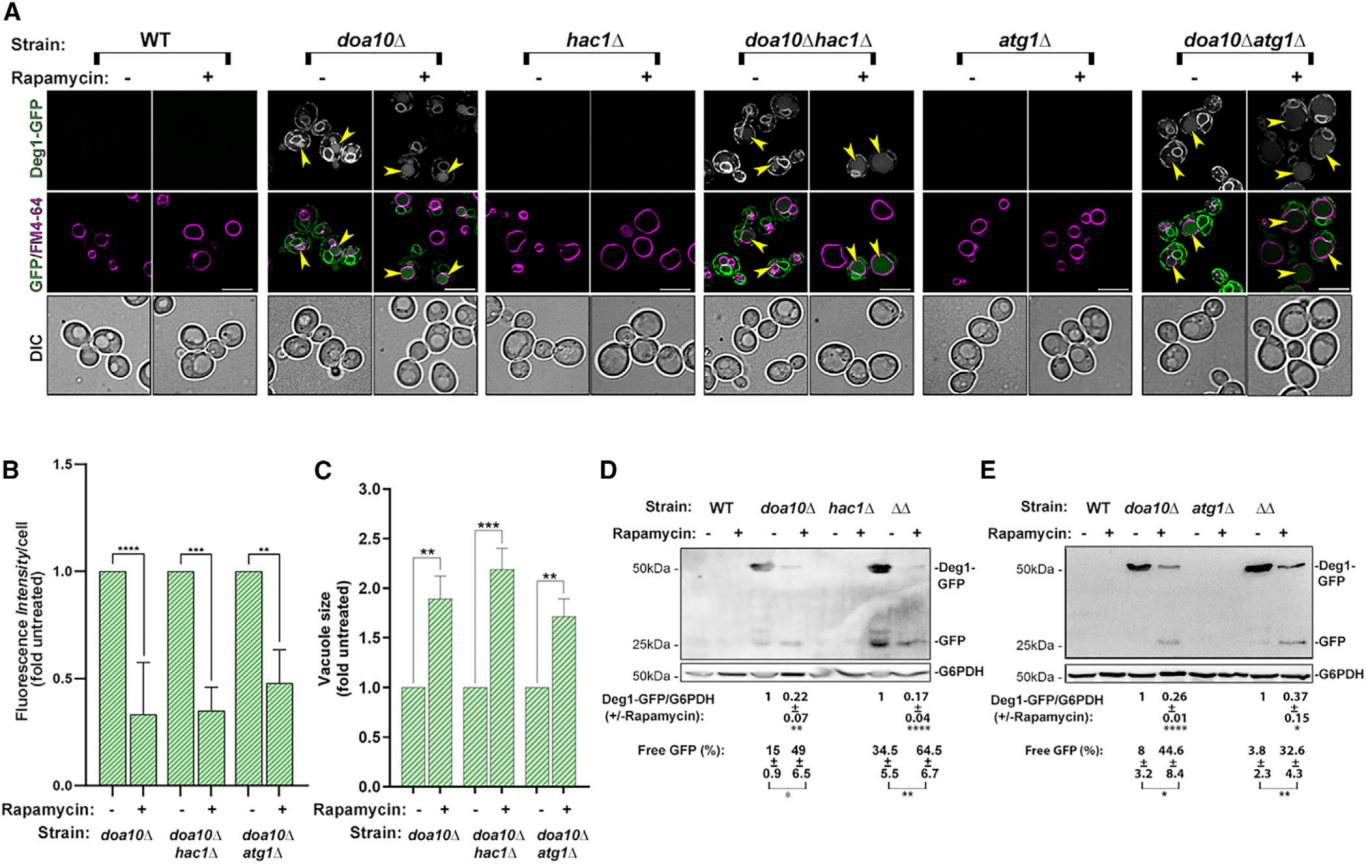
Rapamycin induces Deg1-Vma12-GFP clearance in a UPR- and macro-autophagy-independent manner Six strains (WT, *doa10Δ*, *hac1Δ*, *doa10Δ hac1Δ*, *atg1Δ*, and *doa10Δ atg1Δ*; NSY1962, NSY2017, NSY1963, NSY2073, NSY2120, and NYS2121, respectively) expressing Deg1-Vma12-GFP were grown without (—) or with rapamycin (+, 200 nM for 4 h). The level of Deg1-Vma12-GFP was determined by fluorescence microscopy. Lysosomal membrane was stained by FM4–64 (30 min) (A and B) and immunoblot analysis (D and E), and vacuolar size by fluorescence microscopy (C). (A) From top to bottom: strain, growth condition, GFP, GFP + FM4–64 merge, and differential interference contrast (DIC). Arrowheads point to vacuoles with GFP. Scale bar, 10 μm. (B and C) Quantification of GFP fluorescence per cell (B) and lysosome size (C) in cells from (A) as fold change from untreated cells (set to 1). (D and E) Immunoblots of cell lysates from strains used in A (*hac1Δ*, D; *atg1Δ*, E). From top to bottom: strain (ΔΔ, relevant double deletion), growth conditions (—/+ rapamycin), quantification of Deg1-GFP (corrected by the loading control, and compared between the same culture before and after rapamycin), ± and significance, quantification of free GFP % of total GFP in the same lane, and ± and significance. Free-GFP band is seen after rapamycin treatment in both *doa10Δ* and *doa10Δ atg1Δ* mutant cells. Independence of macro-autophagy proteins is shown in *atg1Δ* (this figure), *atg9Δ*, and *atg7Δ* (Figure S1). Error bars and ± represent mean ± SD; ns, not significant; *p < 0.05, **p < 0.01, ***p < 0.001, ****p < 0.0001. Results represent three independent experiments.

To determine the effect of nutritional stress on the ERAD-C substrate Deg1-Vma12-GFP, WT (WT) and *doa10Δ* mutant cells expressing it were treated with rapamycin. The level of the cargo was assessed by fluorescence microscopy and immunoblot analyses before and after rapamycin treatment. During normal growth, Deg1-Vma12-GFP accumulates in *doa10Δ* mutant cells but not WT cells.^14^ Addition of rapamycin resulted in ~70% reduction of Deg1-Vma12-GFP level in these cells by fluorescence, and immunoblot analysis shows similar reduction in the Deg1-Vma12-GFP level and a significant increase in the level of free GFP (Figure 1). Thus, the majority of the ERAD cargo that accumulates during normal growth in mutant cells deficient in Doa10 can be cleared under nutritional stress. In the following experiments, the effect of rapamycin in cells defective in UPR or autophagy was compared to *doa10Δ* mutant cells.

Independence on UPR was tested in *hac1Δ* mutant cells, in which UPR is abolished. Clearance of Deg1-Vma12-GFP after treatment with rapamycin occurs to the same level in the *doa10Δ* and *doa10Δ hac1Δ* double-mutant cells (Figure 1, not significant). Thus, nutritional-stress-induced clearance of Deg1-Vma12-GFP is independent on UPR induction. Independence on macro-autophagy was tested in mutant cells depleted for the macro-autophagy core components Atg1, Atg9, and Atg7. As we previously reported for several autophagy-defective mutant cells,^15^ there is no accumulation of Deg1-Vma12-GFP in *atg9Δ* single-mutant cells, and it also does not accumulate in *atg1Δ* and *atg7Δ* mutant cells. However, Deg1-Vma12-GFP accumulates in *doa10Δ atg1Δ*, *doa10Δ atg9Δ*, and *doa10Δ atg7Δ* double-mutant cells during normal growth. Importantly, the ability of rapamycin to elicit clearance of Deg1-Vma12-GFP is not dependent on macro-autophagy because the level of the ERAD-C cargo is decreased in *doa10Δ atg1Δ*, *doa10Δ atg9Δ*, and *doa10Δ atg7Δ* double-mutant cells treated with rapamycin as determined by microscopy and immunoblot analyses (Figures 1 and S1). In agreement, immunoblot analysis shows that a similar level of a free GFP is released from Deg1-Vma12-GFP after rapamycin treatment in *doa10Δ* (ATG-WT), *doa10Δ atg1Δ*, *doa10Δ atg9Δ*, and *doa10Δ atg7Δ* mutant cells (Figures 1E, S1E, and S1F). This contrasts with the free GFP band released from the normal ER-membrane protein Rtn1-GFP only in WT but not *atg1Δ* mutant cells (Mochida et al.,^23^ and see Figures S6A–S6C). Thus, rapamycin-dependent clearance of an ERAD-C cargo occurs is independent of macroautophagy.

##### Clearance in the lysosome.

**Figure 2.**
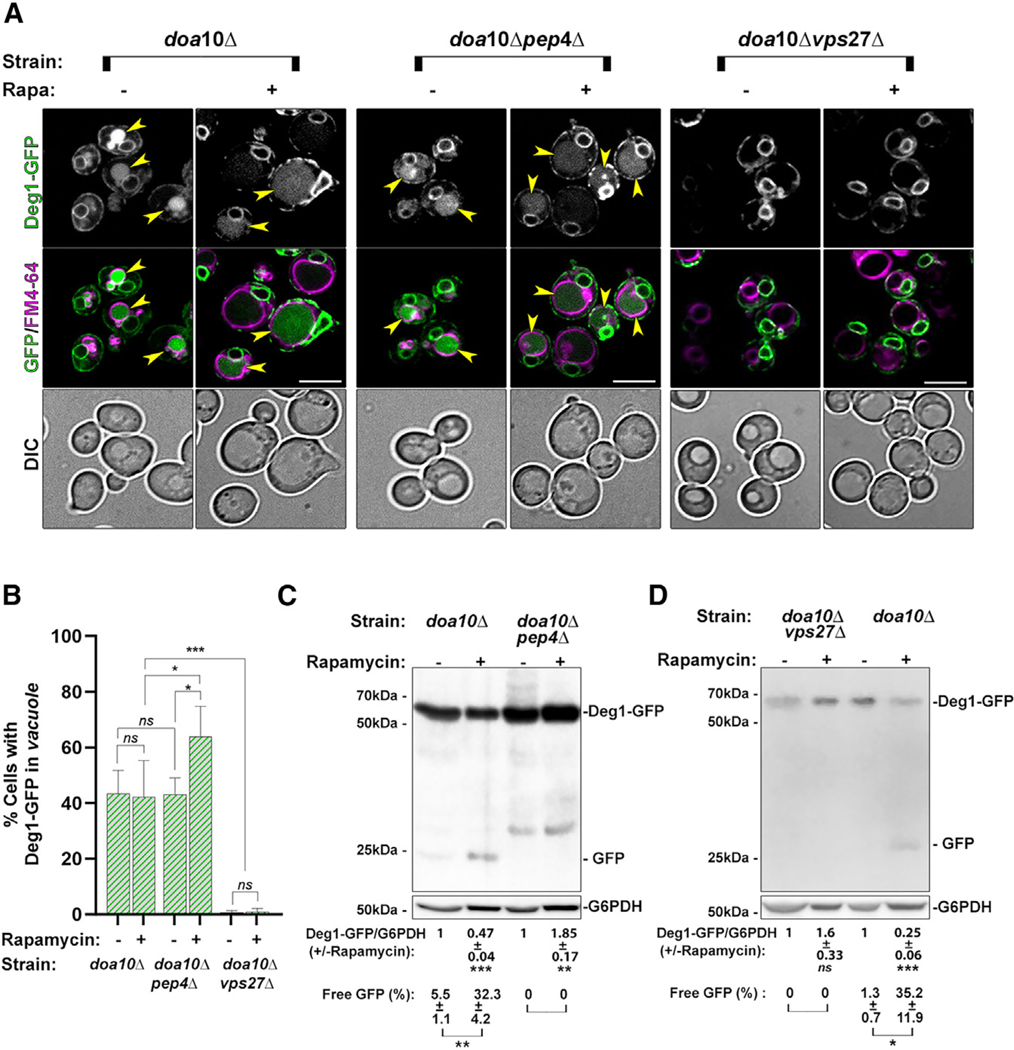
Rapamycin induces an ERAD-C cargo clearance in the lysosome in an ESCRT-dependent manner Three strains, *doa10Δ*, *doa10Δ pep4Δ*, and *doa10Δ vps27Δ* (NSY2017, NSY2123, and NSY2137, respectively) expressing Deg1-Vma12-GFP were grown in regular medium (—) or with rapamycin (+) and stained with FM4–64 and analyzed as described for Figure 1: fluorescence microscopy (A and B) and immunoblot analysis (C and D). (A) From top to bottom: strain, growth condition, GFP, GFP + FM4–64 merge, and DIC. Scale bars, 10 μm. (B) Quantification of cells from (A): % cells with GFP fluorescence in their vacuole. (C and D) Immunoblots of cell lysates from strains used in (A) (*pep4Δ*, C; *vps27Δ*, D). Shown from left to right: (C) *doa10Δ* and *doa10Δ pep4Δ*, and (D) *doa10Δ vps27Δ* and *doa10Δ* (double mutant is on the left). From top to bottom, error bars, and significance as in Figure 1. Results represent four and three independent experiments for microscopy and western blot (WB), respectively. Error bars and ± represent mean ± SD; ns, not significant; *p < 0.05, **p < 0.01, ***p < 0.001, ****p < 0.0001. Results represent three independent experiments.

Deg1-Vma12-GFP can be seen in the lysosomes of (~40%) *doa10Δ* mutant cells even during normal growth, and this number does not change after rapamycin treatment (Figures 2A and 2B). Moreover, under rapamycin treatment, the vacuoles that contain Deg1-Vma12-GFP in *doa10Δ* mutant cells are significantly bigger than those seen during normal growth (Figures 1A and 1C). To support the idea that under nutritional stress clearance of Deg1-Vma12-GFP occurs in the lysosome, we used *pep4Δ* mutant cells defective in vacuolar proteases. Under nutritional stress (rapamycin) using microscopy, more *doa10Δ pep4Δ* double-mutant cells accumulate Deg1-Vma12-GFP in their lysosome (increase from 40% to 60%). Immunoblot analysis shows that the level of Deg1-Vma12-GFP is not decreased but is increased (85% more), and free GFP is not increased upon rapamycin treatment in *doa10Δ pep4Δ* when compared to *doa10Δ* mutant cells (Figures 2A–2C). Thus, microscopy and immunoblot analyses show that during rapamycin treatment, Deg1-Vma12-GFP is delivered to the lysosome of both *doa10Δ* and *doa10Δ pep4Δ* mutant cells but is only degraded in *doa10Δ* mutant cells and not in *doa10Δ pep4Δ* double-mutant cells.

##### Dependence on ESCRT.

ESCRT complexes (endosomal sorting complexes required for transport) catalyze membrane fission events, such as the one needed for direct engulfment of cargo by the lysosomal membrane.^27^ Degradation in the lysosome in an Atg-independent manner suggest that the clearance occurs through micro-autophagy, which is in turn dependent on the ESCRT complexes.^23^ To determine whether ESCRT is required for clearance of the ERAD-C cargo Deg1-Vma12-GFP to reach the lysosome, we used a deletion of the ESCRT subunit, Vps27.^28^ Whereas Deg1-Vma12-GFP accumulates in the vacuole of *doa10Δ* mutant cells (~40%), no Deg1-GFP is seen in the vacuole of *doa10Δ vps27Δ* double-mutant cells with or without rapamycin. In agreement, using immunoblot analysis, the level of Deg1-GFP does not decrease and free GFP is not increased in *doa10Δ vps27Δ* double-mutant cells after treatment with rapamycin. Moreover, whereas free GFP accumulates in *doa10Δ* mutant cells (~35% of the total GFP), it cannot be seen in *doa10Δ vps27Δ* double-mutant cells (Figures 2A, 2B, and 2D). Together, the microscopy and immunoblot analyses show that under rapamycin treatment Deg1-Vma12-GFP is not delivered to the lysosome and is not degraded in *doa10Δ vps27Δ* double-mutant cells. Therefore, ESCRT is needed for delivery of Deg1-Vma12-GFP for clearance under nutritional stress, supporting the idea that it is delivered to the lysosome by micro-autophagy.

##### Dependence on the E3 ubiquitin ligase Rsp5 and its Ssh4 adaptor.

**Figure 3.**
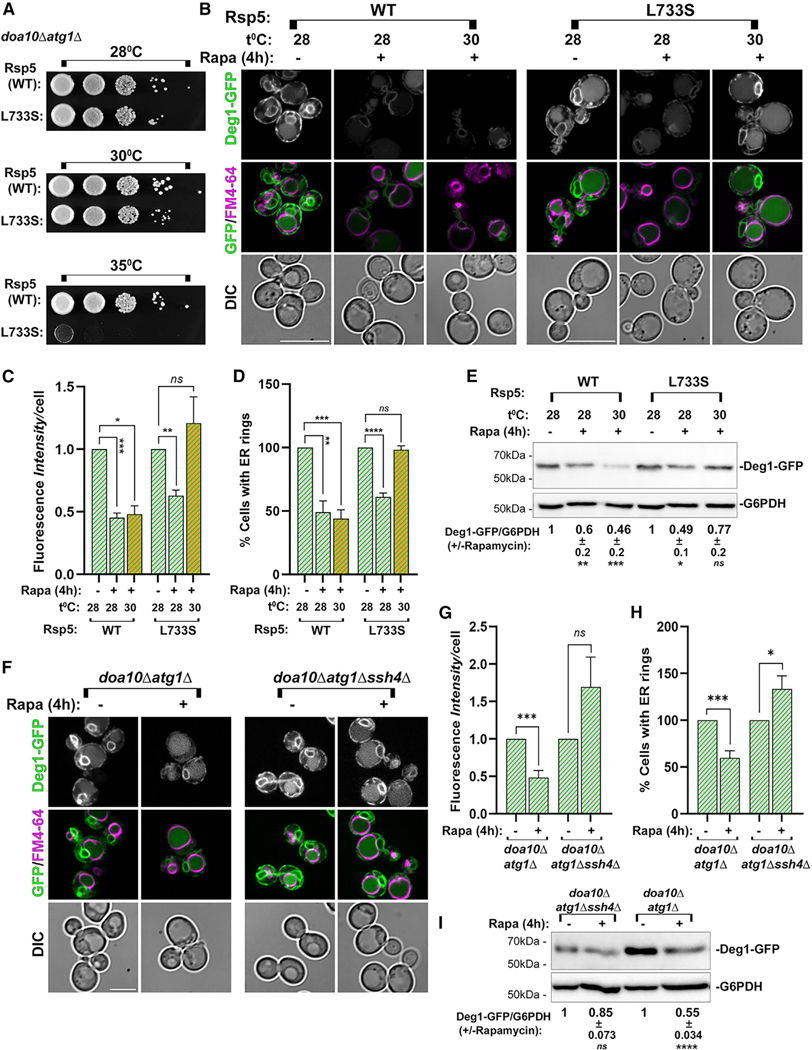
Rapamycin induces an ERAD-C cargo clearance in an Ssh4-Rsp5-dependent manner (A–E) Dependence on the E3 ubiquitin ligase Rsp5. Two strains expressing Deg1-Vma12-GFP were used: Rsp5-WT (NSY2167: *doa10Δ atg1Δ rsp5Δ* + *RSP5-WT*) and Rsp5-L733S (NSY2169: *doa10Δ atg1Δ*, *rsp5Δ* + *RSP5-L733S*). (A) Rsp5-L733S cells (bottom) exhibit a temperature-sensitive growth phenotype at 35^◦^C and a partial growth defect at 30^◦^C (compared to WT, top). Cells were plated on YPD medium (1/10 serial dilution from left to right), and plates were incubated at 28^◦^C, 30^◦^C, or 35^◦^C for 24 h. (B) Cells grown at 28^◦^C in regular medium (—) or with rapamycin added (+) were incubated at 28^◦^C or 30^◦^C, stained with FM4–64, and analyzed as described for Figure 1. Left: *RSP5-WT*; right: *RSP5-L733S*. From top to bottom: strain, growth condition: temperature and —/+ rapamycin, GFP, GFP + FM4–64 merge, and DIC. Scale bars, 10 μm. (C) Quantification of GFP fluorescence per cell (in cells from B) as fold change from untreated cells (set to 1). (D) Bar graph showing quantification of cells with a clear GFP-ring (ER) (in cells from B). (E) Immunoblot of cell lysates from strains used in (B). From top to bottom: strain, growth conditions: temperature, rapamycin (−/+), GFP blot showing Deg1-GFP and G6PDH blot (loading control), quantification of Deg1-GFP, ±, and significance (as described for Figure 1). (F–I) Dependence on the Rsp5 adaptor Ssh4. Two strains expressing Deg1-Vma12-GFP were used: Ssh4-WT (NSY2121: *doa10Δ atg1Δ*); and *ssh4Δ* (NSY2174: *doa10Δ atg1Δ ssh4Δ*). (F) Cells were grown, treated, and analyzed by fluorescence microscopy as in Figure 1: Left: *RSP5-WT*; right: *ssh4Δ*. From top to bottom: strain, growth condition: temperature and −/+ rapamycin, GFP, GFP + FM4–64 merge, and DIC. Scale bars, 10 μm. (G) Quantification of GFP fluorescence per cell (cells from F) as fold change from untreated cells (set to 1). (H) Quantification of cells with a clear GFP-ring (ER) (cells from F). (I) Immunoblot of cell lysates from strains in (F). From top to bottom: strain, growth conditions: rapamycin (−/+), GFP blot showing Deg1-GFP and G6PDH blot (loading control), quantification of Deg1-GFP, ±, and significance (as described for Figure 1). Error bars, significance, and number of independent experiments as in Figure 1. Error bars and ± represent mean ± SD; ns, not significant; *p < 0.05, **p < 0.01, ***p < 0.001, ****p < 0.0001. Results represent three independent experiments.

The ESCRT complexes act as receptors for ubiquitinated membrane proteins to be delivered to the vacuole.^29^ The Rsp5 E3 ubiquitin ligase was shown to associate with ESCRT-0^30^ and to be induced by rapamycin.^31^ Therefore, we tested whether the mechanism by which the accumulated Deg1-Vma12-GFP is delivered to the lysosome depends on its ubiquitination by Rsp5. Because *RSP5* is an essential gene, we used the Rps5-L733S temperature-sensitive (ts) mutant.^32^ Cells, *doa10Δ atg1Δ* expressing and accumulating Deg1-Vma12-GFP, were transformed with a plasmid expressing either WT-Rsp5 or Rsp5-L733S. The endogenous *RSP5* gene was then deleted, yielding cells that depend on Rsp5 expressed from the plasmid, the WT-Rsp5, or Rsp5-L733S (partial growth defect at 30^◦^C and complete block at 35^◦^C, Figure 3A). The effect of rapamycin at 28^◦^C and 30^◦^C on the level of Deg1-Vma12-GFP was assessed by fluorescence microcopy and immunoblot analyses. In cells expressing WT-Rsp5, the level of Deg1-Vma12-GFP fluorescence, the percentage of cells with clear ER-ring (microscopy), and the level of Deg1-Vma12-GFP protein (immunoblot) were significantly lower after rapamycin treatment both at 28^◦^C and 30^◦^C. In contrast, in Rsp5-L733S cells, while at 28^◦^C the levels went down (as in WT cells), during rapamycin treatment at 30^◦^C they remained at the level before the rapamycin treatment (Figures 3B–3E). These results indicate that the E3 ubiquitin ligase Rsp5 plays a role in the clearance of Deg1-Vma12-GFP via micro-ER-phagy.

The Rsp5 E3 ligase catalyzes the attachment of ubiquitin to cargoes recognized by its multiple adaptors.^33^ The Rsp5 adaptor Ssh4 functions under rapamycin treatment to downregulate lysosomal membrane proteins via ESCRT-mediated micro-autophagy.^34^ Therefore, we tested whether Ssh4 is required for clearance of accumulated Deg1-Vma12-GFP. *SSH4*, not required for cell viability, was deleted in *doa10Δ atg1Δ* cells expressing and accumulating Deg1-Vma12-GFP, and the effect of rapamycin on the level of Deg1-Vma12-GFP was assessed. Whereas in *SSH4* WT the level of Deg1-Vma12-GFP fluorescence, the percentage of cells with clear ER-ring (microscopy), and the level of Deg1-Vma12-GFP protein (immunoblot) were significantly lower after rapamycin treatment, in *ssh4Δ* mutant cells they remained at the level before the rapamycin treatment (Figures 3F–3I). These results indicate that the E3 ubiquitin-ligase Rsp5 adaptor Ssh4 plays a role in the clearance of Deg1-Vma12-GFP via micro-ER-phagy, implying that Ssh4 recognizes this aberrant cargo to be ubiquitinated by Rsp5.

The three hallmarks of degradation by micro-autophagy in yeast cells are that it occurs in the lysosome and is independent of the macroautophagy core machinery, but is dependent on the ESCRT complex.^13^ Our findings that during rapamycin treatment Deg1-Vma12-GFP is cleared in the lysosome via a macroautophagy-independent but ESCRT- and Rsp5/Ssh4-dependent manner define the clearance pathway as micro-ER-phagy.

#### Dynamics of rapamycin-induced micro-ER-phagy of an ERAD cargo

**Figure 4.**
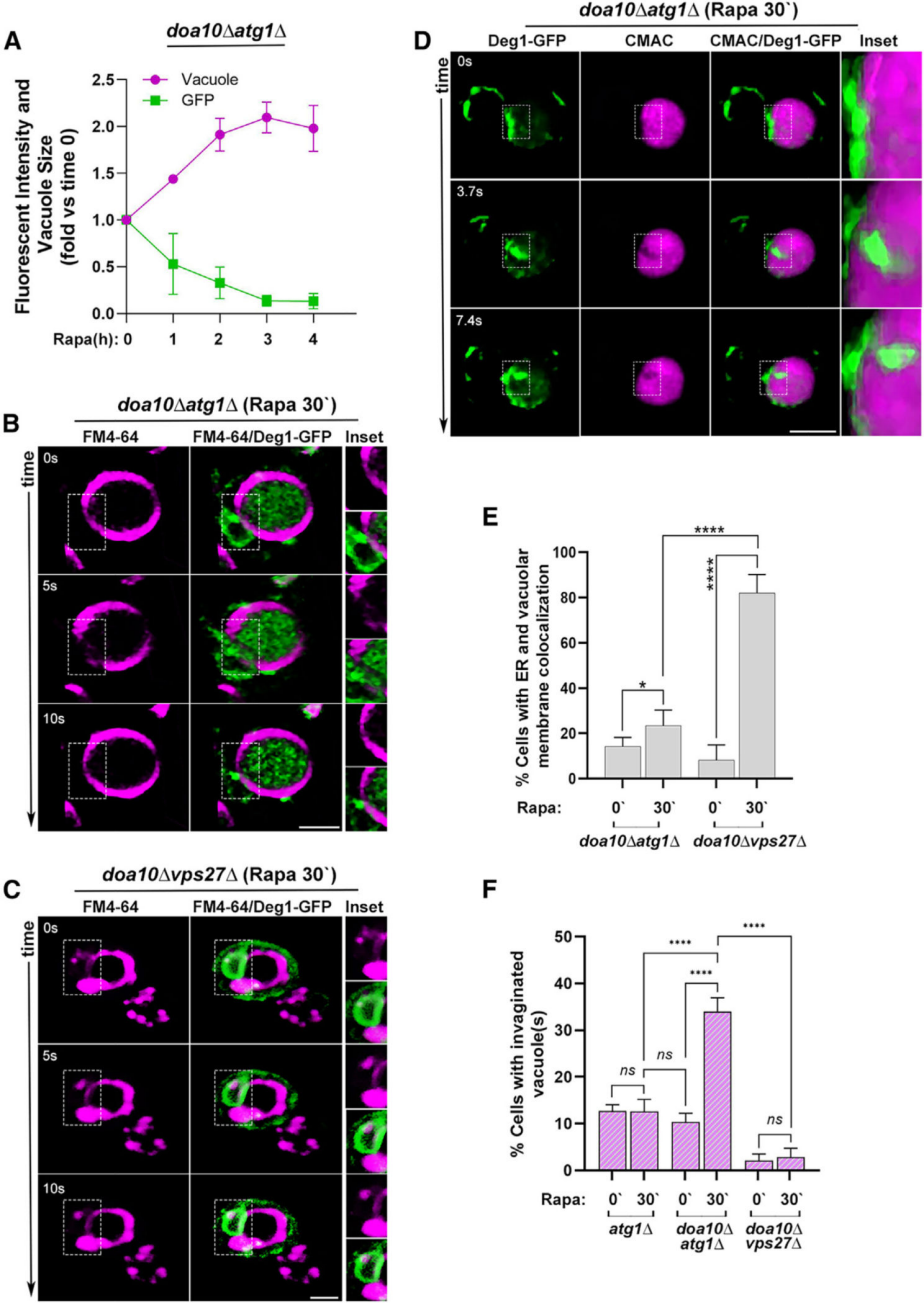
Micro-ER-phagy dynamics Cells that accumulate Deg1-Vma12-GFP during normal growth, *doa10Δ atg1Δ* and *doa10Δ vps27Δ* (NSY2121 and NSY2137, respectively), were incubated with rapamycin (200 nM) for indicated times. The lysosomal membrane or lumen was stained with FM4–64 (B–E) or CMAC (F), respectively, before the cells were imaged by live-cell fluorescence microscopy. (A) Kinetics of fluorescence intensity decrease (green) and vacuolar size increase (magenta) during time of incubation are shown in a plot (fold change from time 0). Most of the changes occur during the first 2 h, which was used for experiments in (B)–(F). (B) Dynamics of Deg1-Vma12-GFP entrance to the lysosome in *doa10Δ atg1Δ* after addition of rapamycin (30 min); double laser. Frames from Video S4 (every 5 s, 0.5 mm/section). From top to bottom: strain, fluorophore, and three frames before, during, and after GFP entry to the lysosome (0, 5 s, 10 s, respectively). From left to right: FM4–64, merge (GFP + FM4–64), and enlargement of the inset (FM4–64, top, to show membrane; merge, bottom, to show entering cargo). (C) Dynamics of the lysosomal membrane in *doa10Δ vps27Δ* mutant cells after addition of rapamycin (30 min); double laser. Frames from Video S8 (every 5 s, 0.5 μm/section; see arrangement in B). Vacuolar membrane is static, and GFP stays near it. (D) Dynamics of Deg1-Vma12-GFP entrance to the lysosome labeled with CMAC in *doa10Δ atg1Δ* after addition of rapamycin (30 min); fast acquisition: double laser with multi-path filter. Frames from Video S12 (every 3.7 s, 0.25 μm/section). From top to bottom: strain, fluorophore, and three frames before, during, and after GFP entry to the lysosome (0, 3.7 s, 7.4 s, respectively). From left to right: GFP, CMAC, merge (GFP + CMAC), and enlargement of the inset from merge (see also Figure S3). (E) Accumulation of Deg1-Vma12-GFP colocalizing with the vacuolar membrane in *doa10Δ atg1Δ* (left) and *doa10Δ vps27Δ* (right) without (0) and with rapamycin (30 min). Shown is percentage of cells in which GFP and vacuolar membrane (FM4–64) co-localize (seen as white in the merge, see Figure S2) in a 90-s video (representative videos without and with rapamycin: *doa10Δ atg1Δ*, Videos S1 and S3; *doa10Δ vps27Δ*, Videos S5 and S7, respectively). (F) Frequency of micro-autophagy events in *atg1Δ* (no Deg1-Vma12-GFP accumulation), *doa10Δ atg1Δ* (left) and *doa10Δ vps27Δ* (right) without (0) and with rapamycin (30 min). Shown is percentage of cells with at least one invagination event in a 90-s video (representative videos: *atg1Δ*, Videos S13, S14, S15, and S16; *doa10Δ atg1Δ*, Videos S1 and S3; *doa10Δ vps27Δ*, Videos S5 and S7). Error bars and ± represent mean ± SD; ns, not significant; *p < 0.05, **p < 0.01, ***p < 0.001, ****p < 0.0001. Results represent three independent experiments. Scale bars, 2 μm.

As shown above, more than 50% of Deg1-Vma12-GFP that accumulates during normal growth is cleared in the lysosomes of *doa10Δ atg1Δ* mutant cells after 4-h incubation with rapamycin. Further analysis showed that cargo clearance and increase of lysosome size starts 30 min after the addition of rapamycin, and most of it occurs within 2 h (Figure 4A). Therefore, we followed the dynamics of individual events of delivery to the vacuole 30 min after addition of rapamycin.

To characterize the dynamics of rapamycin-induced clearance of membrane proteins, we used time-lapse 3D confocal fluorescence microscopy. *doa10Δ atg1Δ* mutant cells were compared before and 30 min after addition of rapamycin (as represented in Videos S1, S2, S3, and S4). These videos were taken with two lasers, every 5 s with 5 μm/optical section z stacks. Only after addition of rapamycin does the vacuolar membrane become very dynamic, and Deg1-Vma12-GFP entrance to the lysosome can be observed in multiple cells (during 90-s videos, e.g., Video S3), and in many cases empty vacuoles became filled with GFP during the video (Figure S2B). Individual events are shown in 3D-reconstruction videos alternating with and without green fluorescence at three different time points: before, during, and after GFP entrance to the vacuole (Video S4 and Figure 4B). In contrast, very few such events can be seen in these cells before addition of rapamycin (Videos S1, and S2; Figure S2A). Importantly, in *doa10Δ vps27Δ* mutant cells defective in ESCRT function that do not clear Deg1-Vma12-GFP under nutritional stress (see above), the vacuolar membrane is static either before or after addition of rapamycin (Videos S5, S6, S7, and S8; Figures S2C, S2D, and 4C). To increase the temporal and special resolutions, time-lapse 3D analysis was conducted for *doa10Δ atg1Δ* mutant cells after treatment with rapamycin (30 min) using two different methods: First, using a single laser to follow the dynamics of the lysosomal membrane labeled by FM4–64, videos of z stacks were taken with 2-fold better temporal and spatial resolution (every 2.5 s, 0.25 μm/optical section) (Video S9 and Figure S3A). Double-laser images were captured at the beginning and end of the videos to show the presence of Deg1-Vma12-GFP in the cells and its entry into the lysosome. Individual membrane invagination events can be clearly seen (Video S10 and Figure S3B) and quantified (see below). Second, using two lasers, CMAC (a dye that labels the lysosomal lumen), and a multi-path filter, we achieved good temporal and spatial resolution (every 3.7 s, 0.25 μm/optical section) (Video S11 and Figure S3C). Using this method, we could clearly observe Deg1-Vma12-GFP entering the lysosome through invaginations (Figure 4D).

The videos were also used for defining micro-ER-phagy pathway steps by quantifying percentage of cells with colocalization of GFP and lysosomal membrane as well as lysosomal membrane invaginations. First, in *doa10Δ vps27Δ* mutant cells defective in micro-autophagy, after addition of rapamycin but not before, the cargo Deg1-Vma12-GFP colocalizes with the lysosomal membrane (compare Videos S1, S3, S5, and S7 and Figures S2A–S2D, colocalization seen as white). Quantification of this colocalization reveals that before the addition of rapamycin, colocalization of cargo with the lysosomal membrane is very low in both *doa10Δ atg1Δ* and *doa10Δ vps27Δ* mutant cells (Videos S1 and S5; Figures S2A and S2C). Interestingly, after addition of rapamycin, whereas in *doa10Δ atg1Δ* mutant cells there is only a modest increase in cells with such colocalization (because the GFP enters the lysosome), in >80% of the *doa10Δ vps27Δ* mutant cells the GFP colocalizes with the lysosomal membrane (Figure 4E). Second, quantification of cells with invaginations in the lysosomal membrane showed a >3-fold increase in cells with vacuolar membrane invaginations under rapamycin treatment in *doa10Δ atg1Δ* cells that accumulate Deg1-Vma12-GFP, but not in *atg1Δ* cells that do not accumulate it; no invaginations were seen in *doa10Δ vps27Δ* that accumulate Deg1-Vma12-GFP (Figure 4F; Videos S13, S14, S15, and S16). This indicates that the presence of accumulated cargo is needed for rapamycin-induced lysosomal membrane invaginations. Together, these analyses separated two independent steps of the micro-ER-phagy pathway: rapamycin-dependent cargo colocalization with the lysosomal membrane and ESCRT-dependent cargo engulfment and entry into the lysosome.

The speed of individual micro-ER-phagy events in *doa10Δ atg1Δ* mutant cells after the addition of rapamycin (30 min) was determined from the videos taken with the three methods: double lasers (5-s intervals, e.g., Video S3), single laser (2.5-s intervals, e.g., Video S9), and double lasers with the multi-path filter (3.7-s intervals, e.g., Video S11). The determined speeds were ~13, 7.5, and 9 s, respectively. The three methods were similar in respect of the percentage of cells that showed invaginations (~30%) and the rate of invaginations (~10^−3^ event/s/cell, *p* value between each two of the three methods not significant). This supports the idea that these three analyses follow the same invagination events. Because the temporal and special resolutions are better with the latter two methods, we suggest that the former is an overestimate and that individual micro-ER-phagy events take ~8s (Table S1).

These results show that engulfment of ER domains containing the aberrant membrane protein Deg1-Vma12-GFP occurs via the lysosomal membrane early during rapamycin treatment, it requires sequential induction by rapamycin and the ESCRT complex, and individual events occur within seconds.

#### Clearance of macro-ER-phagy cargoes during nutritional stress

**Figure 5.**
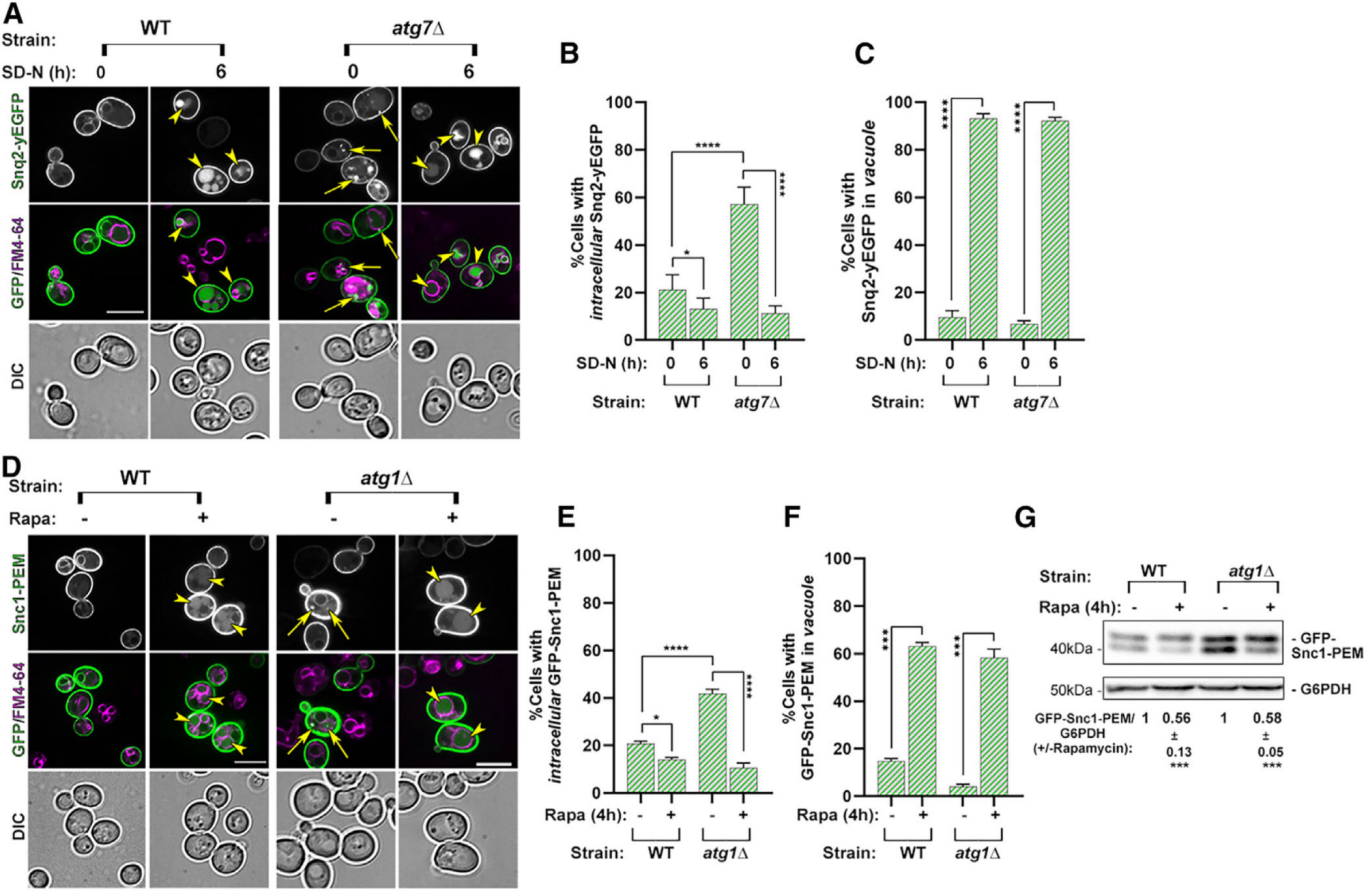
Nutritional stress induces clearance of macro-ER-phagy cargoes via micro-ER-phagy (A–C) Clearance of overexpressed Snq2-GFP during nitrogen starvation using microscopy analysis. (A) Two strains, WT *and atg7Δ* (NSY825 and NSY1894, respectively) were transformed with a plasmid to overexpress Snq2-GFP (pNS1507) and grown in regular medium (time 0) or shifted to medium without nitrogen (SD-N, for 6 h). The lysosomal membrane was stained with FM4–64, and cells were visualized by live-cell confocal microcopy. From top to bottom: strain, medium (+/− N), Snq2-yEGFP, merge of GFP and FM4–64, and DIC. Arrows point to intracellular Snq2-yEGFP and arrowheads to GFP inside the vacuole. Scale bar, 10 μm. (B and C) Quantification of cells from (A): percentage of cells with aberrant intracellular Snq2-GFP (B) and with Snq2-GFP inside the vacuole (C). (D–G) Clearance of overexpressed GFP-Snc1-PEM under rapamycin treatment using microscopy (D–F) and immunoblot analyses (G). (D) Two strains, WT and *atg1Δ* (NSY825 and NSY1567, respectively) were transformed with a plasmid to overexpress GFP-Snc1-PEM (pNS1407) and grown in regular medium (−) or with rapamycin (+). Cells were stained with FM4–64 and visualized by live-cell confocal microcopy. From top to bottom: strain, rapamycin (−/+), GFP-Snc1-PEM, merge of GFP and FM4–64, and DIC. Arrows point to intracellular GFP-Snc1-PEM and arrowheads to GFP inside the vacuole. Scale bars, 10 μm. (E and F) Bar graphs show percentage of cells (from D) with aberrant intracellular GFP-Snc1-PEM (E) and with GFP-Snc1-PEM inside the vacuole (F) in cells. (G) Immunoblot analysis: Cell lysates from (D) were used for immunoblot analysis using anti-GFP antibodies. From top to bottom: strain, treatment (−/+ rapamycin), GFP blot, G6PDH blot, quantification of GFP-Snc1-PEM (as described for Figure 1), ±, and significance. Molecular weight (MW) markers on the left; analyzed proteins on the right (G6PDH, loading control). Microscopy and immunoblot analyses show that clearance of excess ER-membrane proteins occurs in an Atg-independent manner (Atg7 and Atg1) in the lysosome and is ESCRT dependent (Figures S4 and S5). Error bars represent mean ± SD; ns, not significant; *p < 0.05, **p < 0.01, ***p < 0.001, ****p < 0.0001. Results represent three independent experiments.

To determine the effect of nutritional stress on the macro-ER-phagy substrates, we tested the effect of two nutritional stress inducers, nitrogen starvation (6 h) and rapamycin (200 nM, 4 h), on two macro-ER-phagy cargoes, Snq2-GFP and GFP-Snc1-PEM, overexpressed in WT cells. Using microscopy, the percentage of cells that contain intracellular ER cargoes (not in the vacuole), although low, decreased, while the percentage of cells in the cargoes in the vacuole clearly increased. In agreement, immunoblot analysis using GFP-Snc1-PEM showed that its level significantly increased during nutritional stress (Figures 5 and S4).

To determine the independence on core Atgs, the effect of nutritional stress on Snq2-GFP and GFP-Snc1-PEM clearance in mutant cells depleted of two core Atg components, Atg1 and Atg7, was compared to that in WT cells. Most of the intracellular macro-ER-phagy cargoes that accumulate in *atg7Δ* and *atg1Δ* mutant cells during normal growth are efficiently cleared under nutritional stress. First, using microscopy, when *atg7Δ* mutant cells expressing Snq2-GFP are shifted to medium without nitrogen, most of the cells lose the intracellular Snq2-GFP that is not in the vacuole (Figures 5A and 5B). Second, most *atg1Δ* and *atg7Δ* mutant cells that accumulate GFP-Snc1-PEM during normal growth clear intracellular GFP-Snc1-PEM (not present in the vacuole) during treatment with rapamycin, as seen by microscopy (Figures 5D, 5E, S4A, and S4B). Moreover, the level of GFP-Snc1-PEM protein is significantly lower after treatment of WT, *atg1Δ*. and *atg7Δ* cells with rapamycin, as seen by immunoblot analysis (Figures 5G, S4D, and S4E, respectively). In addition, we have previously shown that Ypt1 guanosine triphosphatase (GTPase) is required for macro-ER-phagy, and *ypt1–1* mutant cells accumulate macro-ER-phagy cargoes under normal growth conditions.^35^ Trs85 is a subunit of the autophagy-specific guanine nucleotide exchange factor for Ypt1,^36^ and its deletion is expected to mimic depletion of Ypt1 in macro-ER-phagy.^35^ Here we show that rapamycin treatment can also clear GFP-Snc1-PEM from *ypt1–1* mutant cells (Figures S5A and S5B), and nitrogen starvation can help clear Snq2-GFP, which accumulates in *trs85Δ* mutant cells during normal growth (Figures S5D and S5E). This clearance is robust because under nutritional stress intracellular macro-ER-phagy cargoes, GFP-Snc1-PEM and Snq2-GFP, are cleared from >70% of *atg7Δ* and *atg1Δ* mutant cells, as seen by microscopy (Figures 5C and S4C). The ~50% clearance of GFP-Snc1-PEM seen by immunoblot analysis is an underestimate, because most of this protein resides on the plasma membrane (and cannot be internalized).^37^ Together, these results indicate that nutritional stress, rapamycin, or nitrogen starvation induces robust clearance of multiple ER-phagy cargoes in a macro-autophagy-independent manner.

An outlier among the core Atgs with respect to micro-ER-phagy is Atg17.^38^ We have previously observed that while intracellular GFP-Snc1-PEM does not accumulate in *atg17Δ* mutant cells during normal growth (because, unlike other core Atgs, Atg17 is not required for constitutive autophagy), it accumulates in SD-N medium (Lipatova et al.^15^; Figures S5G and S5H). Here, using microscopy and immunoblot analyses, we show that during rapamycin treatment while some GFP-Snc1-PEM can be seen in the vacuole in *atg17Δ* mutant cells, the delivery to the vacuole is lower than in WT and *atg7Δ* mutant cells and the level of the cargo is not decreased (Figures S5G–S5J). Thus, clearance of GFP-Snc1-PEM under nutritional stress partially relies on Atg17 but not other core autophagy machinery, e.g., Atg1, Atg7, and Ypt1.

Clearance of macro-ER-phagy cargoes under nutritional stress occurs in the lysosome. First, under nutritional stress, GFP-Snc1-PEM can be seen in the vacuole of >50% WT, *atg1Δ*, and *atg7Δ* mutant cells (Figures 5 and S4), and Snq2-GFP can be seen in the vacuole of ~90% WT and *atg7Δ* mutant cells (Figures 5A and 5C). GFP-Snc1-PEM and Snq2-GFP are also delivered to the vacuole in *ypt1–1* and *trs85Δ* mutant cells upon rapamycin treatment or nitrogen starvation, respectively (Figures S5A–S5F). To further confirm that this clearance occurs in the vacuole, we used the *pep4Δ* mutation that results in defective vacuolar proteases. If the cargo is delivered to the vacuole but not degraded, we expect to see an increase in the number of cells that accumulate it in their vacuole. In *pep4Δ* single-mutant and *atg7Δ pep4Δ* double-mutant cells, >80% cells accumulate GFP-Snc1-PEM in the vacuole (compared to ~50% in WT and *atg7Δ* cells) (Figures S4A–S4C). Immunoblot analysis supports this idea (Figure S4D).

Micro-autophagy in yeast was shown to be dependent on the ESCRT complex.^39^ To determine whether clearance of GFP-Snc1-PEM requires ESCRT, we used *vps27Δ* mutant cells. Intracellular GFP-Snc1-PEM does not accumulate in *vps27Δ* mutant cells during normal growth while it accumulates in *atg7Δ* and *atg7Δ vpas27*D, indicating that Vps27 is not required for constitutive macro-ER-phagy. Importantly, under rapamycin treatment, whereas intracellular GFP-Snc1-PEM is cleared on *atg7Δ* mutant cells, it is not cleared in *atg7Δ vps27Δ* mutant cells. Finally, while GFP-Snc1-PEM accumulates in the vacuole of >50% of WT and *atg7Δ* mutant cells, almost no GFP-Snc1-PEM reaches the vacuoles of *vps27Δ* and *atg7Δ vps27Δ* mutant cells during normal growth or under nutritional stress (Figures S4A–S4C). In agreement, immunoblot analysis shows that the level of GFP-Snc1-PEM is not decreased in *vps27Δ* and *atg7Δ vps27Δ* mutant cells under nutritional stress (Figure S4E). Thus, under nutritional stress the ESCRT complex is required for clearance of an ER-phagy cargo in the lysosome.

In summary, the degradation of macro-ER-phagy fluorescent cargoes under nutritional starvation is shown by five independent criteria: First, by microscopy, a lower level of intracellular cargo structures; second, by microscopy, a dramatic increase of fluorescence in the vacuole; third, by immunoblot analysis, a lower level; fourth, dependence on Pep4, a lysosomal protease; and fifth, dependence on ESCRT (by microscopy and immunoblot analyses). Together these results show that, like the accumulated ERAD-C cargo, macro-ER-phagy cargoes are cleared under nutritional stress (>70%; rapamycin or nitrogen starvation) in the lysosome in a macro-autophagy-independent but ESCRT-dependent manner, defining the clearance pathway as micro-ER-phagy.

#### Clearance of normal ER and mitochondrial membrane proteins under nutritional stress

The “normal” ER-membrane proteins, Sec63-GFP, Hmg1-GFP, and Rtn1-GFP, expressed from their promoters, were shown to be substrates for macro-ER-phagy upon rapamycin treatment. Specifically, after 12–24 h of treatment with rapamycin (200 ng/mL), a free GFP band is observed by immunoblot analysis and GFP was seen in the lysosomes of WT (not quantified) but not *atg1Δ* mutant cells.^23^ We wanted to confirm that in our hands a normal ER-membrane protein is not degraded by the micro-ER-phagy (macro-autophagy independent) that degrades aberrant membrane proteins after a short treatment with rapamycin (200 nM, 4 h). First, as previously published, upon rapamycin treatment (4–24 h), Rtn1-GFP expressed from its endogenous locus is degraded to show a free GFP band in WT, but not in *atg1Δ* mutant cells (Figure S6A). This result agrees with the previously published (albeit unquantified) data.^23^ Second, when Rtn1-GFP is expressed from its endogenous locus, after 4 h GFP can be seen in the lysosome of a very small fraction of WT cells (~10%) and in more after 16 h (40%). Clearly, there is no GFP in the lysosomes of *atg1Δ* mutant cells even after 16 h (Figures S6B and S6C). This contrasts with the massive delivery of aberrant proteins to lysosomes of *atg1Δ* mutant cells after 4 h of the same rapamycin treatment. Thus, normal membrane proteins are not cleared via the nutritional-stress-induced micro-ER-phagy that clears aberrant membrane proteins we discovered here. Moreover, while clearance of most of the aberrant membrane proteins by micro-ER-phagy occurs in the first 2 h of the nutritional stress, degradation of normal membrane proteins by macro-ER-phagy occurs later, between 4 and 24 h.

To determine whether other membrane proteins in excess can be cleared during nutritional stress, we tested mitochondrial proteins, which can be cleared under oxidative or nutritional stresses.^40^ The inner mitochondrial marker Cox4-mCherry (containing the mitochondria-targeting sequence of Cox4 fused to mCherry and expressed from ADH1, a high-expression constitutive promoter) was expressed in WT and in mutant cells defective in macro-autophagy, *atg1Δ* and *atg7Δ*. Delivery of the mitochondria to the vacuole (labeled with CMAC) is observed only in WT cells, not in *atg1Δ* or *atg7Δ* mutant cells, after 4–24 h of treatment with rapamycin (Figures S6D and S6E). Thus, whereas mitochondrial proteins can be cleared later by macro-autophagy, they are not cleared by nutritional-stress-induced micro-ER-phagy.

#### Summary

Using different kinds of nutritional stresses, multiple cargoes, and different assays, our results indicate that a nutritional-stress-induced micro-ER-phagy pathway selectively clears aberrant ER-membrane proteins but not normal ER-membrane or excess mitochondrial proteins. It depends on Ssh4-Rsp5 E3 ubiquitin ligase and ESCRT and occurs earlier than degradation of normal membrane proteins by macro-ER-phagy, and its individual events occur within seconds.

### The effect of ER stress and heat on UPR on ERAD-C and ER-phagy cargoes

**Figure 6.**
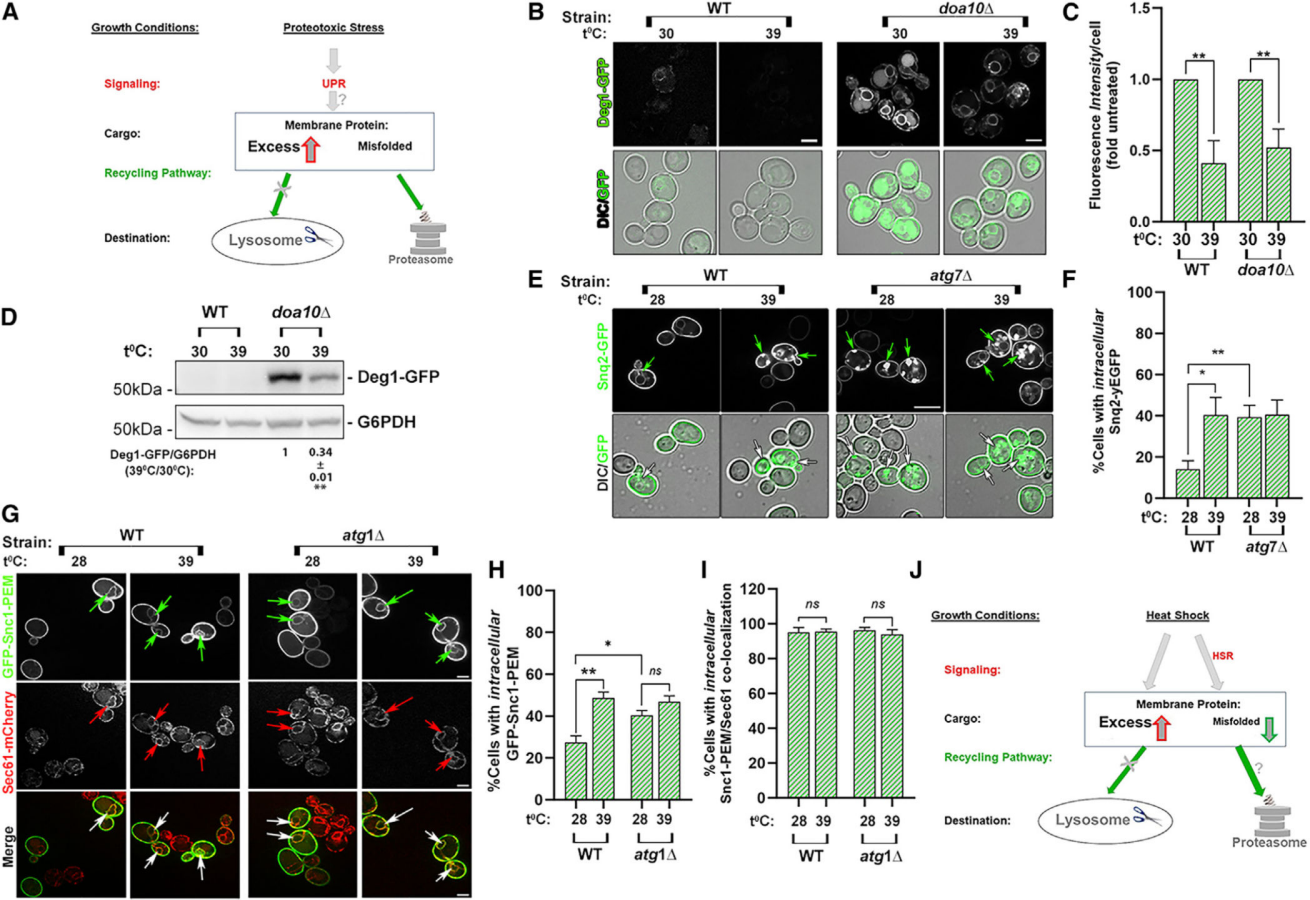
Effects of ER and heat stresses on ERAD-C and macro-ER-phagy cargoes (A) Model summarizing the effect of ER stress on aberrant membrane proteins. ER stress causes accumulation of GFP-Snc1-PEM in WT cells (results shown in Figure S7), while it has no effect on the level of Deg1-GFP, an ERAD-C cargo.^41^ (B–J) Heat stress has opposite effects on ERAD-C and macro-ER-phagy cargoes. (B–D) Heat shock helps clear Deg1-Vma12-GFP. (B) WT (left) and *doa10Δ* mutant (right) cells expressing Deg1-Vma12-GFP (NSY1962 and NSY2017, respectively) were grown at 30^◦^C or shifted to 39^◦^C for 1 h and tested by live-cell microscopy. From top to bottom: strain, growth temperature, GFP fluorescence, and GFP + DIC merge. (C) Quantification of Deg1-GFP fluorescence intensity/cell from cells described in (B). (D) Immunoblot analysis. Lysates from cells from (B) were tested by immunoblot analysis using anti-GFP antibodies to detect GFP-Snc1-PEM (top) and anti-G6PDH antibodies (loading control). From left to right: WT cells at 30^◦^C and 39^◦^C, and *doa10Δ* mutant cells at 30^◦^C and 39^◦^C (MW markers shown on the left). From top to bottom: strain, growth temperature (28^◦^C or 30^◦^C), blot, quantification of Deg1-GFP (corrected by the loading control, and compared between the same culture before and after the temperature shift), ±, and significance. (E–I) Heat shock causes accumulation of macro-ER-phagy cargoes. (E and F) Heat causes accumulation of Snq2-yEGFP. (E) WT and *atg7Δ* mutant cells (NSY825 and NSY1894, respectively) expressing Snq2-yEGFP from a plasmid (pNS1507) were grown at 28^◦^C or shifted to 39^◦^C (1 h). From top to bottom: strain, growth temperature (28^◦^C or 39^◦^C), GFP fluorescence, and DIC/GFP merge. Arrows point to intracellular Snq2-yEGFP. Scale bar, 10 μm. (F) Quantification of intracellular Snq2-yEGFP in cells from E (%). (G–I) Heat causes accumulation of GFP-Snc1-PEM in the ER. (G) WT and *atg1Δ* mutant cells expressing the ER marker Sec61-mCherry from its endogenous promoter (NSY1649 and NSY1653, respectively) and overexpressing of GFP-Snc1-PEM from a plasmid (pNS1407) were grown at 28^◦^C or shifted to 39^◦^C (1 h) before testing by live-cell microscopy. From top to bottom: strain, growth temperature (28^◦^C or 39^◦^C), GFP fluorescence, mCherry fluorescence, and merge. Arrows point to intracellular GFP-Snc1-PEM colocalizing with Sec61-mCherry. Scale bars, 5 μm. (H) Quantification of cells with intracellular GFP-Snc1-PEM (%). (I) Quantification of cells (from G) in which internal GFP-Snc1-PEM colocalizes with Sec61 (%). Error bars, significance, and independent experiments as in Figure 1. (J) Model summarizing the effect of heat shock and HSR on aberrant membrane proteins (see also Figure S8). Heat shock, through HSR, helps clear the ERAD cargo Deg1-GFP accumulated in *doa10Δ* mutant cells (downward green arrow) probably by the proteasome (question mark) because HSR sends misfolded proteins for degradation in the proteasome.^71^ In contrast, heat (not through HSR) exacerbates the accumulation of GFP-Snc1-PEM and Snq2-GFP in WT cells (upward red arrow). Error bars and ± represent mean ± SD; ns, not significant; *p < 0.05, **p < 0.01, ***p < 0.001, ****p < 0.0001. Results represent three independent experiments.

While ER stress induces UPR and clearance of ERAD cargo in general,^24^ it was previously shown that applying ER stress to cells that express the ERAD-C cargo Deg1-Vma12-GFP does not affect its clearance in mutant cells defective in ERAD-C.^41^ To determine the effect of ER stress on accumulation of the ER-phagy cargo GFP-Snc1-PEM, WT cells expressing GFP-Snc1-PEM were treated with tunicamycin, and the effect on accumulation of GFP-Snc1-PEM in the ER was determined using fluorescence microscopy. Tunicamycin treatment, which induced UPR as confirmed using a β-galactosidase assay, resulted in a significant (~2-fold) increase in GFP-Snc1-PEM that accumulated in the ER of WT cells, which do not accumulate this cargo during normal growth (Figure S7). Thus, ER stress does not help clear an ERAD-C cargo in mutant cells defective in the ERAD-C-specific ubiquitin ligase and causes further accumulation of a macro-ER-phagy cargo (Figure 6A).

To explore the possible relationship between HSR and ERAD-C, we determined the effect of HSR induction by heat on Deg1-Vma12-GFP levels in WT and *doa10Δ* mutant cells grown at 30^◦^C and 39^◦^C (1 h) using live-cell fluorescence microscopy and immunoblot analyses. Using microscopy, very little Deg1-GFP accumulation is observed in WT cells at 30^◦^C, and it is significantly lower at 39^◦^C (using immunoblot analysis, the Deg1-Vma12-GFP band is below the level of detection in WT cells). In *doa10Δ* mutant cells defective in ERAD response, >50% Deg1-Vma12-GFP is cleared at 39^◦^C compared to 30^◦^C, as shown by fluorescence microscopy and immunoblot analyses (Figures 6B–6D). Therefore, exposing cells to a heat shock, when the HSR is induced, helps remove the ERAD-C cargo Deg1-GFP that accumulates in *doa10Δ* mutant cells during normal growth. To determine whether HSR is involved in clearing ERAD-C cargo, we tested whether induction of HSR without the heat shock can help clear ER-phagy cargo. HSR was induced by expressing an activated heat shock factor 1, Hsf1-R206S,^42^ at normal growth temperature, 30^◦^C. Expression of Hsf1-R206S in WT and *doa10Δ* mutant cells expressing Deg1-Vma12-GFP resulted in about 10-fold activation using a LacZ HSR assay (about half of the activation caused by 39^◦^C for 1 h; Figure S11E). Hsf1-R206S expression of in *doa10Δ* mutant cells resulted in a significant decrease of Deg1-Vma12-GFP levels (microscopy 36% and immunoblot 71%) (Figures S8A–S8C). These results show that induction of the HSR either by heat or by expression of activated Hsf1 helps clear the ERAD-C cargo Deg1-Vma12-GFP that accumulates in *doa10Δ* mutant cells.

The effect of heat on the intracellular accumulation of the ER-phagy cargo Snq2-GFP was determined in WT and *atg7Δ* mutant cells by comparing between cells grown at 28^◦^C and 39^◦^C (1 h) using live-cell fluorescence microscopy. After a heat shock, more WT cells (2.5-fold) accumulate intracellular Snq2-GFP, similar to the accumulation of these structures in *atg7Δ* mutant cells at both 28^◦^C and 39^◦^C (Figures 6E and 6F). This is true also for a second ER-phagy cargo, GFP-Snc1-PEM, which accumulates inside autophagy-defective mutant cells and co-localizes with the ER marker Sec61.^35^ Intracellular accumulation of GFP-Snc1-PEM in WT and *atg1Δ* mutant cells expressing Sec61-mCherry was compared between cells grown at 28^◦^C and 39^◦^C (1 h) using live-cell fluorescence microscopy. In WT cells, most of the GFP-Snc1-PEM accumulates on the plasma membrane, and very few cells accumulate inside at 28^◦^C. After incubation of the cells at 39^◦^C (1 h), a significantly higher percentage of the cells accumulates GFP-Snc1-PEM inside the cells and colocalizes with Sec61-mCherry. In *atg1Δ* mutant cells defective in the ER-phagy response, as previously published,^35^ ~2-fold more GFP-Snc1-PEM accumulates at 28^◦^C when compared to WT cells, and this does not change significantly at 39^◦^C (Figures 6G–6I). Therefore, exposing cells to a heat shock results in an increase in the intracellular accumulation of two ER-phagy cargoes, Snq2 and GFP-Snc1-PEM, in the ER of WT cells. To determine whether the effect of the heat shock on accumulation of GFP-Snc1-PEM in WT cells depends on HSR induction, we tested whether induction of HSR without heat has a similar effect. HSR induced by expressing an activated heat shock factor 1, Hsf1-R206S, at normal growth temperature, 28^◦^C (as described above) in either WT or *atg7Δ* mutant cells had no effect on the accumulation of GFP-Snc1-PEM (Figures S8D and S8E). Thus, the adverse effect of heat on clearance of the ER-phagy cargo GFP-Snc1-PEM is not mediated by the HSR.

#### Summary

Heat has opposite effects on ERAD-C and macro-ER-phagy cargoes, helping clear the first through induction of HSR and exacerbating the accumulation of the second independently of HSR (Figure 6J).

### Stress signaling and clearance of ER-membrane proteins during normal growth

#### UPR signaling and ERAD-C cargo clearance

The following reports suggested that UPR is not needed for ERAD-C: deletion of *IRE1*, which is required for UPR induction, did not affect the level of another ERAD-C cargo, Ubc6^43^; and in a screen for deletion mutants that accumulate Deg1-Ura3, a Doa10 substrate, neither *hac1Δ* nor *ire1Δ*, which are required for UPR, were identified.^14^ Because the first report cited “data not shown” and the second described a negative result, we determined here whether UPR is needed for clearance of the ERAD-C substrate Deg1-Vma12-GFP. First, UPR is not induced in *doa10Δ* mutant cells in which Deg1-Vma12-GFP accumulates or in WT cells in which it is efficiently cleared (it is ~10-fold induced by tunicamycin). Second, we used *hac1Δ* cells, in which UPR is abolished,^44^ and abolishment was confirmed in *hac1Δ* and *hac1Δ doa10Δ* cells expressing Deg1-Vma12-GFP upon addition of tunicamycin. Because the detected level of Deg1-Vma12-GFP is very low in *hac1Δ* mutant cells, we confirmed that it is expressed in these cells using two approaches: Deg1-Vma12-GFP level is increased in *hac1Δ doa10Δ* double-mutant cells when compared to *hac1Δ* single-mutant cells, and in *hac1Δ* mutant cells (and WT cells) treated with MG132, a proteasomal inhibitor. Neither *hac1Δ* nor *hac1Δ doa10Δ* mutant cells accumulate more Deg1-Vma12-GFP as seen by microscopy and immunoblot analyses (in fact it seems lower) (Figure S9). These results show that under normal growth conditions, clearance of the misfolded membrane protein Deg1-Vma12-GFP by ERAD-C in WT cells does not require the UPR signaling pathway, and its accumulation in mutant cells defective in ERAD (*doa10Δ*) does not induce UPR signaling.

#### TORC1 signaling and macro-ER-phagy cargo clearance

We have previously shown that shuttling of a single membrane protein in excess for degradation in the lysosome via macro-ER-phagy during normal growth utilizes the conserved macro-autophagy machinery components.^6^ However, based on several criteria, stress-induced autophagy is not stimulated in WT cells overexpressing and clearing a macro-ER-phagy cargo or in mutants defective in this process and accumulating this cargo. For example, there was no elevation in the Atg8 protein level and no increase in a quantitative starvation-induced autophagy assay in WT or mutant cells overexpressing the macro-ER-phagy cargo GFP-Snc1-PEM.^15^ Here, we wished to determine whether TORC1 signaling, which couples cell growth and metabolism,^45^ plays a role in this process. The TORC1 kinase is active under normal growth conditions but is inactive under nutritional stress or after addition of the drug rapamycin.^24^ Because TORC1 is essential for cell viability (and cannot be deleted), to determine the possible role of TORC1 signaling in macro-ER-phagy, we tested the phosphorylation state of downstream substrates of TORC1. First, phosphorylation of Atg13 by TORC1 inhibits autophagy, while unphosphorylated Atg13 induces autophagy under nutritional stress via a known mechanism.^25^ Second, TORC1 kinase phosphorylates Sch9, which in turn promotes cell growth via known targets, whereas dephosphorylation of Sch9 halts cell growth under nutritional stress^46^ (Figure S10A). The phosphorylation state of TORC1 substrates was compared in WT cells with and without overexpression of GFP-Snc1-PEM and in *ypt1–1* mutant cells that accumulate GFP-Snc1-PEM (see Lipatova et al.^15^ and Figure S10B) under normal growth conditions. As a control for TORC1 inhibition, the phosphorylation state of two TORC1 substrates was determined under starvation or upon rapamycin treatment. The phosphorylation state of endogenous Atg13 was tested using immunoblot analysis and anti-Atg13 antibodies, and the phosphorylation state of HA-tagged Sch9 expressed from a plasmid was determined using immunoblot analysis and anti-HA antibodies.

As expected, in WT cells Atg13 is phosphorylated under normal growth conditions (p-Atg13, higher molecular-weight [MW] bands) and dephosphorylated (Atg13, lower band) in cells grown in a medium lacking nitrogen or upon addition of rapamycin.^47^ In contrast, Sch9-HA is phosphorylated under normal growth conditions (pSch9, higher MW band) and dephosphorylated (Sch9, lower band) in cells grown in a medium lacking nitrogen or after addition of rapamycin.^48^ This is true also for *ypt1–1* mutant cells, which are defective in macro-ER-phagy and accumulate GFP-Snc1-PEM (about 4-fold more than WT cells; Figure S10B). Importantly, overexpression of GFP-Snc1-PEM in WT or *ypt1–1* mutant cells did not change the phosphorylation state of Atg13 or Sch9-HA during normal growth conditions and under nitrogen starvation or rapamycin treatment (Figure S10). Thus, under normal growth conditions, TORC1 signaling is not affected either upon clearance of GFP-Snc1-PEM by macro-ER-phagy in WT cells or by its accumulation in mutant cells defective in macro-ER-phagy.

#### Signaling and partial overlap of ER-phagy and ERAD

We have previously shown that an ER-phagy cargo can be partially cleared by ERAD-C in mutants defective in autophagy^15^ and that UPR is induced in autophagy-defective mutant cells that accumulate an ER-phagy cargo.^35^ To determine whether UPR is needed for ERAD-C to clear this ER-phagy cargo, we tested the effect of *hac1Δ*, which abolishes UPR, on the level of GFP-Snc1-PEM in cells that accumulate it. Inhibition of UPR by the *hac1Δ* did not affect the level of GFP-Snc1-PEM indicating that UPR signaling is not needed for ERAD-C to clear an ER-phagy cargo.^15^ We have also shown that an ERAD-C cargo can be cleared by ER-phagy in mutant cells defective in ERAD-C.^15^ The remaining question is whether TORC1 signaling plays a role in ER-phagy clearance of the ERAD cargo Deg1-Vma12-GFP. To address this question, the phosphorylation state of the TORC1 substrate Atg13 was determined in WT and *doa10Δ* mutant cells expressing Deg1-Vma12-GFP using immunoblot analysis and anti-Atg13 antibodies. As expected, in WT cells Atg13 is phosphorylated under normal growth conditions (p-Atg13, higher MW band) and dephosphorylated (Atg13, lower band) in cells after addition of rapamycin.^47^ This is true also for *doa10Δ* mutant cells, which are defective in ERAD. Importantly, expression of Deg1-Vma12-GFP in WT or *doa10Δ* mutant cells did not change the phosphorylation state of Atg13 (Figures S11A and S11B). Thus, TORC1 signaling does not play a role in clearance of the ERAD-C cargo Deg1-Vma12-GFP by ER-phagy in WT cells. Because there is no difference in TORC1 signaling in *doa10Δ* mutant cells when Deg1-Vma12-GFP accumulates, clearance of Deg1-Vma12-GFP by Atg11, Trs85, and Atg9 in *doa10Δ* mutant cells is also not dependent on TORC1 signaling. Together, these results show that when macro-ER-phagy and ERAD clear each other’s cargoes in the respective mutant cells, TORC1 and UPR signaling do not play a role in this partial overlap.

#### HSR signaling

We determined whether HSR is induced when ERAD-C or ER-phagy cargo is expressed in WT cells or accumulates in *doa10Δ* and *atg7Δ* mutant cells, respectively. Induction of HSR was determined using a reporter in which HSR elements, HSEs, are fused to LacZ^49^ and a b-galactosidase assay.^50^ For ERAD-C cargo, WT and *doa10Δ* mutant cells not expressing or expressing Deg1-Vma12-GFP were transformed with a plasmid expressing the HSR reporter. For macro-ER-phagy cargo, WT and *atg7Δ* mutant cells not expressing or expressing GFP-Snc1-PEM were transformed with a plasmid expressing the HSR reporter. Reporter activity was determined in cell lysates,^50^ and activation was verified by showing about 4-fold induction when comparing cells grown at 30^◦^C and 39^◦^C. Under normal growth conditions (30^◦^C), HSR was not induced by expression of Deg1-Vma12-GFP in WT cells or when it accumulated in *doa10Δ* mutant cells (Figure S11C). Similarly, HSR was not induced by expression of GFP-Snc1-PEM in WT cells or when it accumulated in the ER of *atg7Δ* mutant cells (at 28^◦^C^15^) (Figure S11D). Thus, while HSR can be induced at 39^◦^C, at 30^◦^C neither Deg1-Vma12-GFP or GFP-Snc1-PEM clearance (in WT cells) nor their accumulation (in *doa10Δ* or *atg7Δ* mutant cells, respectively) induces the HSR. These results show that HSR is not induced during clearance of either macro-ER-phagy or ERAD cargoes or when these cargoes accumulate in macro-ER-phagy or ERAD-defective mutant cells.

#### Summary

Under normal growth conditions, UPR, TORC1, and HSR signaling are not induced during clearance of ERAD-C and macro-ER-phagy cargoes or when these cargoes accumulate in mutant cells defective in ERAD-C or ER-phagy. Thus, both ER-QC pathways, ERAD-C and macro-ER-phagy, clear misfolded and surplus proteins during normal growth in a constitutive manner.

## DISCUSSION

Based on results presented here, we draw three major conclusions: first, nutritional stress is the only cellular stress that leads to clearance of aberrant membrane proteins; second, this clearance occurs via a novel selective micro-ER-phagy pathway delivering aberrant ER-membrane proteins, but not normal ER-or mitochondrial-membrane proteins, for degradation in the lysosome and depends on the E3 ubiquitin ligase Rsp5, its Ssh4 adaptor, and the ESCRT complex; third, ER-QC of aberrant membrane proteins during normal growth by ERAD and macro-ER-phagy is constitutive.

First, we term TORC1-regulated nutritional stress “clean stress” because, unlike proteotoxic and heat stresses, it does not add to the cellular proteotoxic load while clearing aberrant membrane proteins. The effect of two other stresses, ER stress and heat, depends on the cargoes and can range from clearance, through no effect, to additional accumulation of the cargo. A possible reason for the inability of ERAD to clear aberrant proteins is that while it induces UPR it also induces accumulation of additional misfolded proteins, which depend on specific ubiquitin ligases without much overlap.^51^ Heat also induces accumulation of misfolded proteins. Degradation of an ERAD cargo, but not macro-ER-phagy cargoes, by heat-induced HSR is probably done together with the bulk of damaged proteins by proteasomes,^52^ which is in turn increased during heat shock.^53^ This highlights nutritional stress as a way for inducing clearance of aberrant membrane proteins, including disease-related membrane proteins.

Second, we identify and characterize a nutritional-stress-induced micro-ER-phagy pathway selective for clearance of aberrant membrane proteins. Thus far, very little is known about the regulation and mechanisms of micro-autophagy or its cross-talk with macro-autophagy.^13^ Hallmarks of an ER-stress-induced micro-autophagy pathway that delivers expended “ER whorls” in yeast are that it occurs in the lysosome and does not need most of the core Atgs of macro-autophagy, but requires the ESCRT complex.^10^ Using these criteria^12,13^ and live-cell microscopy evidence, we identify the nutritional-stress-induced clearance of aberrant ER-membrane proteins as a micro-ER-phagy pathway. Because unlike macro-autophagy this pathway does not require ATGs and APs, it is more cell economical and can occur earlier (in the first 2 h) than nutritional-stress-induced macro-autophagy (4–24 h), which degrades a small fraction of multiple normal proteins, cytoplasmic and membranal, and parts of organelles, such as mitochondria, via APs.^54^

**Figure 7.**
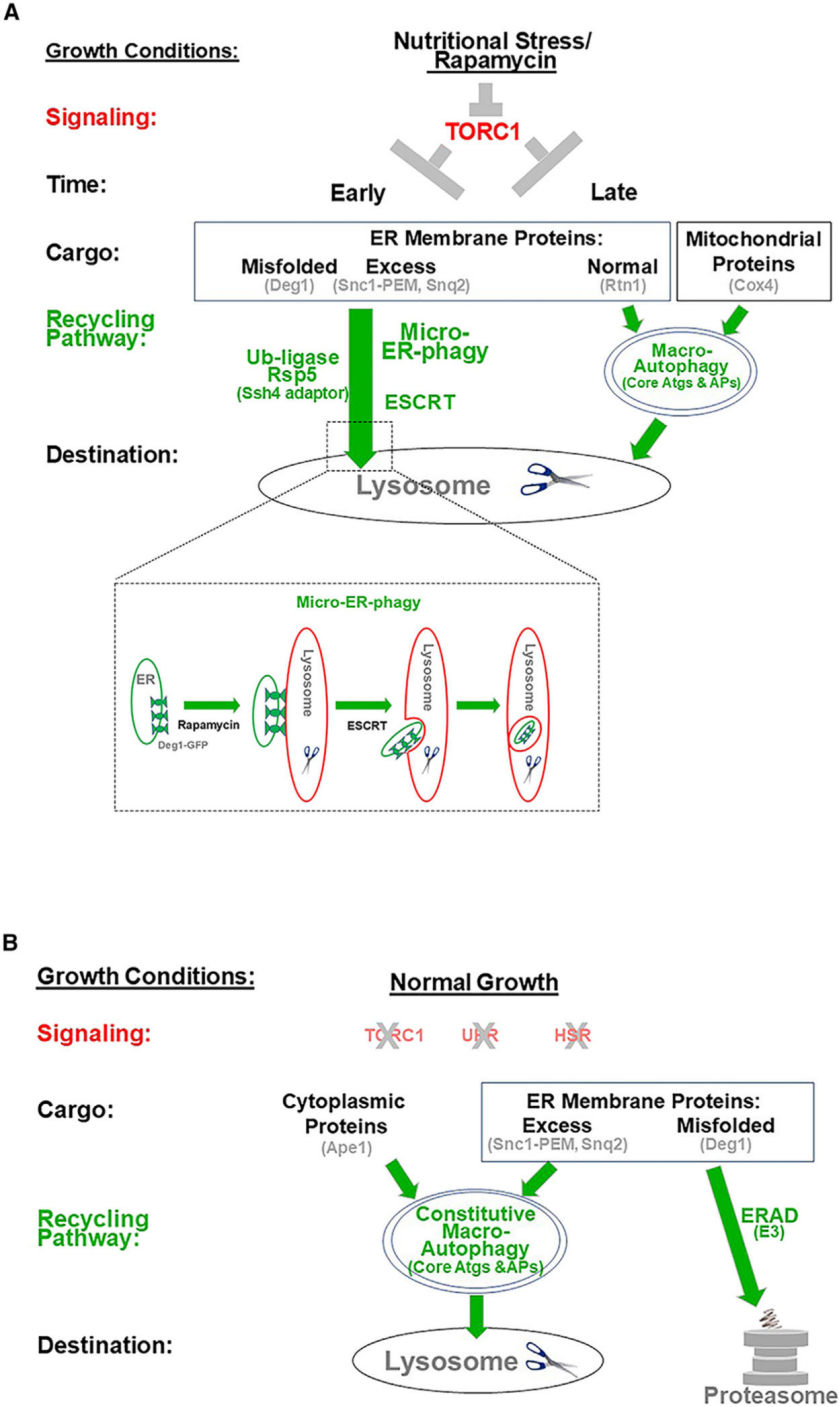
Models for recycling of aberrant membrane proteins during normal growth and under nutritional stress (A) Model for nutritional-stress-induced and TORC1-regulated micro-ER-phagy pathway. Under nutritional stress (nitrogen starvation or rapamycin treatment), TORC1 kinase inhibition results in induction of macro- and micro-autophagy. Macro-autophagy delivers cytoplasmic proteins (e.g., Ape1^72^) or mitochondria fragments^73^ for degradation in the lysosome. It requires the core Atg proteins and the formation of APs.^64,74^ This pathway can deliver normal ER-membrane proteins expressed from their own promoters (e.g., Rtn1, Hmg1, and Sec63^23^) and mitochondrial proteins to the lysosome. However, only a small fraction of normal membrane proteins is delivered through this pathway; it absolutely depends on the core Atgs and occurs late during the stress (right).^10^ Here we identify a new micro-autophagy pathway for effective clearance of aberrant membrane proteins, misfolded or in excess, that accumulate in mutants defective of ER-QC in the lysosome (left). This pathway does not require the core Atgs, Atg1, Atg7, Atg9, and Ypt1 GTPase and its activator TRAPP III but requires the E3 ubiquitin ligase Rsp5, its Ssh4 adaptor, and the ESCRT complex. Clearance through this pathway occurs much earlier than in the macro-autophagy pathway and is selective for aberrant membrane proteins, misfolded or in excess. Inset: two steps in the micro-ER-phagy pathway of the ERAD cargo Deg1-Vma12-GFP that accumulates in *doa10Δ* mutant cells during normal growth. In the first step, which is dependent on rapamycin, ER loaded with Deg1-Vma12-GFP colocalizes with the lysosomal membrane. In the next step, which is dependent on ESCRT, the Deg1-Vma12-GFP enters the lysosome. (B) Model for constitutive ERAD and macro-ER-phagy during normal growth. Under normal growth conditions, misfolded (e.g., Deg1-Vam12-GFP) and excess (e.g., Snc1-PEM and Snq2) individual membrane proteins are cleared effectively in WT cells by ERAD or macro-ER-phagy, respectively, but accumulate in cells defective in their respective pathway. The three protein-quality-control signaling pathways (red), TORC1, UPR, and HSR, are not induced when such cargoes are expressed (gray X represents no role), neither in WT cells where they are cleared nor in mutant cells where they accumulate (Figures S9–S11).

Cargo selectivity of the new pathway is supported by the fact that both normal ER membrane and excess mitochondrial proteins are not cleared by it but, instead, they are degraded by macro-ER-phagy, which is induced much later and is dependent on core Atgs (Mochida and Nakatogawa^10^; Kanki et al.^55^; Figure S6). Moreover, while lysosomal membrane invaginations were observed in cells that accumulate aberrant membrane proteins (*doa10Δ*), they were not seen in cells that do not accumulate this cargo (*DOA10*-WT, Figure 4F). This highlights this new pathway as a therapeutic target for clearance of disease-related membrane proteins (Figure 7A).

The results presented here point to three mechanistic insights of the new pathway. (1) Dependence of the clearance on the E3 ligase Rsp5 suggests that ubiquitination plays a role in the selective sorting of the cargo. (2) While the exact mechanism of cargo recognition by E3 ligases is not clear,^33^ it is known that Rsp5 needs adaptors for such recognition. We identified Ssh4 as the Rsp5 adaptor for recognition of the Deg1-Vma12-GFP cargo. (3) Dependence of the clearance on ESCRT suggest that ESCRT complexes play a role both in sorting the ubiquitinated cargo, its engulfment by the lysosomal membrane, and the membrane fission event, which is the last step of the internalization of the cargo into the lysosome. These three machinery components are also required for a micro-autophagy pathway that degrades normal lysosomal membrane proteins.^34^ In contrast, the micro-ER-phagy pathway we identified here does not degrade normal ER-membrane proteins.

We propose that micro-ER-phagy is superior to macro-ER-phagy and ERAD for efficient clearing early during nutritional stress. (1) It is the most economical process among the three. Macro-ER-phagy requires multiple selective cargo receptors and formation of autophagosomes, and it sends normal proteins for degradation.^10,11^ Likewise, ERAD requires specific ubiquitin ligases for different cargoes, their extraction from membranes, and degradation to proteasomes,^8^ which costs energy (~80 ATPs/molecule) and is slow (~13 s/molecule).^56^ (2) It can clear whole domains of ER loaded with aberrant proteins in ~8 s. (3) Clearance of unwanted cellular components, such as aberrant membrane proteins, should happen early during the stress, whereas valuable cellular components should be degraded only if the stress persists.

We show for the first time the dynamics of individual micro-ER-phagy events using live-cell fluorescence microscopy. The idea that micro-autophagy occurs via invaginations of the lysosomal membrane has been supported by electron microscopy, which provides the special resolution required for visualizing such events without time sequence.^39,57^ Previous live-cell fluorescence microscopy studies of micro-autophagy lacked either temporal and special resolution^39^ or special information.^57^ Using time-lapse 3D confocal microscopy, we documented individual events of ER-domain encapsulation with enough temporal and special resolution. Macro-ER-phagy events were excluded by using mutant cells defective in this process (*doa10Δ atg1Δ*), and negative controls included normal growth (no rapamycin), cells that do not accumulate aberrant membrane proteins (*DOA10 atg1Δ*), and mutant cells defective in ESCRT (*doa10Δ vps27Δ*). From these experiments we draw the following conclusions: (1) individual events occur in ~8 s; (2) we can clearly observe GFP-labeled cargo inside magenta-labeled lysosomal membrane or luminal invaginations (using two lasers, 5 s and 3.7 s resolution) and, acquiring images at double speed (using single laser, 2.5 s), we show that opening and closing invagination events occur within ~7 s; and (3) we identified two independent steps in the micro-ER-phagy pathway: rapamycin-dependent localization of the cargo to the vicinity of the lysosomal membrane and ESCRT-dependent engulfment of the cargo by the lysosomal membrane (Figure 7A, inset). To our knowledge, these data provide the best support for micro-autophagy occurrence through lysosomal membrane invagination, the first documentation of live individual micro-autophagy events, and the first separation of cargo delivery to the lysosomal membrane from cargo engulfment by the lysosomal membrane.

Third, while ERAD, including ERAD-C, which clears misfolded membrane proteins during normal growth, was characterized decades ago,^8^ we characterized macro-ER-phagy of excess individual membrane proteins more recently.^6,15^ Regardless, the involvement of stress signaling during normal growth in either process is currently unknown. The TORC1, UPR, and HSR signaling pathways play major roles in clearance of excess or damaged cellular components under nutritional, proteotoxic, and heat stresses, respectively.^58,59^ Here, we show that none of the known signaling pathways—TORC1, UPR, or HSR—is induced or required for efficiently clearing excess or misfolded single membrane proteins by ERAD-C or ER-phagy, respectively, defining the ER-QC pathways that clear aberrant cargoes from the ER under normal growth conditions as constitutive (Figure 7B).

### Future questions

Currently, not much is known about mechanisms specific to micro-autophagy in general.^13^ Important new questions our study inspires concern mechanisms underlying the specific sorting and clearance of aberrant proteins by the nutritional-stress-induced micro-ER-phagy pathway. For example, the type of ubiquitination used for cargo sorting and engulfment by the E3 ubiquitin ligase Rsp5, its Ssh4 adaptor, and the membrane fission complex ESCRT, whether aberrant ubiquitinated proteins are sorted to ER-membrane domains excluding normal proteins, which ESCRT complex subunit(s) recognize the marked cargo, and which are important for cargo engulfment and lysosomal membrane fission. In addition, the machinery components we identified here play roles in other processes and pathways. For example, ESCRT plays a role in micro-autophagy and closure of APs in macro-autophagy in yeast and human cells,^60,61^ and Rsp5 plays roles in trafficking, sorting, and degradation of multiple proteins under different growth conditions.^62^ Open questions include whether there are genes required only for micro- and not macro-autophagy and what machinery is needed for cargo delivery to the vicinity of the lysosomal membrane. Other more general questions raised by our results are first, whether there is crosstalk between TORC1-regulated macro- and micro-autophagy. For example, Atg17 plays a role under nutritional stress in both macro-autophagy^63^ and micro-ER-phagy (shown here). In macro-autophagy, in addition to its known role in autophagosome formation,^63^ we have previously shown that Atg17 acts also in a late step by recruiting ESCRT to autophagosomes.^60^ Does Atg17 play a similar role in micro-autophagy? Second, why do ER and heat stresses have different effects on macro-ER-phagy and ERAD cargoes? Using classical genetics, machinery components and mechanisms of the conserved macro-autophagy were discovered first in yeast.^64^ Advanced approaches in yeast^65^ make it an ideal system for asking questions about micro-autophagy.

### Relevance to human health

The signaling and recycling pathways studied here are conserved from yeast to human cells.^24,25^ Therefore, the relevance of the conclusions stated above to human health and disease should be considered. We reason that recycling pathways of aberrant proteins during normal growth and under stress are even more crucial for the growth of human cells than of yeast cells due to their longer lifespan that allows accumulation of such aberrant proteins. Two extreme cases of aberrant protein accumulation include cancer and neuronal disorders. In cancer, rapid proliferation can compromise QC mechanisms. For example, accumulation of the membrane protein human epidermal growth factor receptor 2 (HER2) in about 20% of breast cancers is associated with a more aggressive disease.^66^ The long-living neuronal cells are especially sensitive to accumulation of aberrant proteins that evade QC. For example, a role for ERAD in modulating the activity of γ-secretase, a membrane protein complex, was shown to regulate the pathology of Alzheimer’s disease.^67^ Indeed, mutations in membrane-embedded proteins and the recycling pathways are implicated in human disease.^68–70^ Therefore, understanding the regulation of constitutive ER-QC pathways and the effect of different stresses on their cargoes in human cells is important for a variety of diseases. In particular, exploring the existence of a selective micro-ER-phagy pathway in human cells and especially identifying factors specific to this pathway might provide a therapeutic target to induce clearance of disease-related proteins.

### Limitations of the study

While in this study we show clearance of multiple misfolded or excess protein cargoes by the micro-ER-phagy pathway, we used one misfolded cargo, Deg1-Vma12-GFP, which under normal growth conditions is degraded through ERAD-C. Additional ERAD cargoes should be tested to make broader conclusions. While 3D live-cell fluorescence microscopy is required for following cargo engulfment by the lysosomal membrane and its entrance to the lysosome, correlated light and electron microscopy can be used for visualization of the membrane(s) involved the site of lysosomal membrane invagination.

## RESOURCE AVAILABILITY

### Lead contact

Requests for further information and resources should be directed to and will be fulfilled by the lead contact, Nava Segev (nava@uic.edu).

### Materials availability

All unique/stable reagents generated in this study are available from the lead contact with a completed materials transfer agreement.

### Data and code availability

All data reported in the paper and any additional information required to analyze the data reported herein are available from the lead contact upon request. This paper does not report original code.

## STAR★METHODS

### EXPERIMENTAL MODEL AND SUBJECT DETAILS

All yeast strains and plasmids were generated using standard techniques and are described. Yeasts were grown in YPD (10 g/L yeast extract, 20 g/L peptone, 2% glucose) or synthetic dropout (SD; 7.1 g/L yeast nitrogen base, 2% glucose, 1x Ammino acid dropout mix) media at 28^◦^C and analyzed in the mid-log phase (OD_600_ 0.5–0.8).

For most strains, gene disruptions and tagging were performed using homologous recombination of PCR-amplified cassettes (69). To generate strains with *atg1Δ*, *atg7Δ*, *atg9Δ*, *pep4Δ*, *vps27Δ*, and *ssh4Δ*, respective target genes were replaced with either *nat*, *hydro*, *or kanMX cassettes from* pAG25, pAG32, *or* pFA6a. All strains were screened for correct insertion of cassettes using diagnostic PCR and selection plates.

For Rsp5 mutants, cells were first transformed with pRS316-Rsp5 WT or PRS316-Rsp5 L733S. The colonies carrying either plasmid were selected on SD-Ura plates. Single colonies carrying the WT or mutant Rsp5 were inoculated into a 250 mL baffled flask with 25 mL SD-Ura at OD600 0.02 and grown overnight at 25^◦^C with shaking. When the culture reached OD_600_ between 0.3 and 0.5, cells were pelleted and resuspended in 50 mL YPD media with 0.5% adenine and grown for 90 min. Cells were transformed with PCR product carrying rps5Δ::NatR selection cassette by heat shock and grown in 1.5 mL of SD-Ura media for 3h. Cells were plated onto YPD plates containing Nat and G418 to select for *rps5Δ* while maintaining *doa10Δ* in the original strain. Mutant cells were confirmed using diagnosis PCR. The presence of either WT and Rsp5 L733S plasmid were confirmed by recovering the plasmid from each respective yeast strain and sequencing the plasmids after transforming and purifying each one from bacteria (Cat #C2987I, New England BioLabs).

### METHOD DETAILS

#### Nutritional stress microscopy

Yeast cells were transformed with the plasmid carrying the gene of interest and plated on selective agar plates. At least six colonies per experiment were selected and assessed using fluorescence microscopy to ensure the plasmid is actively expressing the desired protein. Three colonies with similar levels of GFP or mCherry signal were chosen for further analysis.

Cells were inoculated from single colonies into tubes containing YPD or Selective Media and incubated at 30^◦^C with rotation for approximately 12–16 h. After incubation, the OD_600_ of the cell culture was measured using a BioMATE 160 UV-visible Spectrophotometer and diluted into 35 mL of fresh media. For experiments with cells expressing GFP-Snc1-PEM or Snq2-yEGFP, cells were diluted in 25 mL SD-LEU media. The culture was then incubated at 28^◦^C while shaking for 16 h (overnight) until it reached the mid-log phase 0.5–0.8 OD_600_ for ERAD-C cargo or 1–1.5 OD_600_ for ER-phagy cargo.

Once the desired OD_600_ was reached, the culture was transferred to a new clean flask, treated with 200μM Rapamycin (Cat # R-5000, LC Laboratories) for a final concentration of 0.2μM, and incubated for 4 h at 28^◦^C with shaking. For untreated cells, 500 μL – 1 mL of cell culture was transferred to a new clean tube, treated with FM4–64 (75) (Cat #T3166, Invitrogen) at a final concentration of 1.6μM, and incubated at 28^◦^C with shaking for 30–60 min. For treated cells, 500 μL of cell culture was transferred to a new clean tube, treated with FM4–64 at a final concentration of 1.6μM, and incubated at 28^◦^C with shaking for 30–60 min before the 4-h treatment was completed. Cells were spun down and washed with fresh media to remove excess dye before imaging. Images were acquired using a Yokogawa Spinning Disk confocal Leica DM8i inverted microscope. Fluorescence intensity per cell was compared between untreated and treated cells using ImageJ. For ER-phagy cargoes, accumulation of GFP-Snc1-PEM or Snq2-yEGFP in cells was quantified and expressed as “percent cells with GFP signal inside of cells (not in the vacuole) and inside of vacuole”.

For nitrogen starvation experiments with ER-phagy cargoes, cells were grown overnight from OD600 0.02 in SD-LEU media to 1–1.5 OD_600_. Cells were washed twice with DI water, transferred into 25 mL SD-N (0.17% yeast nitrogen base without amino acid and ammonium sulfate (Cat #Y2030, US Biological), 2% glucose), and starved for 6h at 28^◦^C. Cells were spun down for microscopy, and fluorescent microscopy was performed using a Yokogawa Spinning Disk confocal Leica DM8i inverted microscope. Accumulation of Snq2-yEGFP in cells was quantified and expressed as “percent cells with GFP signal inside-aberrant ER” (not in the vacuole) and percent of cells with GFP signal inside of vacuole.

Cells with Rsp5 mutant were grown in YPD media up to mid-log phase at 28^◦^C before treatment with rapamycin. After adding rapamycin, cells were divided into two flasks and incubated one at 28^◦^C and another at 30^◦^C for 4h. Samples were collected before and after treatment and temperatures shift for microscopy and immunoblot.

#### Immunoblot

Cells expressing Deg1-GFP were collected before and after stress, aiming for a total OD_600_ of 5–7. The samples were then spun down and resuspended in 200 μL of 10% TCA (Cat # BP555–250, Fisher BioReagents) and incubated on ice for 30 min. Samples were spun down. The resulting pellet was washed with cold acetone, spun down again, removed supernatant, and left to dry on the bench for 10 min. Once completely dry, the pellets were resuspended in 100 μL of MURB buffer, and approximately 50 μL of glass beads were added. Samples were vortexed at maximum speed for 5 min in a cold room, then incubated in a water bath at 75^◦^C for 10 min. After the 10-min incubation, the samples were spun down for 1 min at 15,060 rpm to precipitate cell debris and glass beads before loading onto a 10% SDS-PAGE gel. The gel ran for 1 h and 30 min at 125V.

Cells expressing GFP-Snc1-PEM were collected before and after treatment, aiming for a total OD_600_ of 2.5–2.7. The samples were spun down, washed with DI water, kept at −80^◦^C, or processed for total lysate isolation using 0.1 N NaOH. The pellet was resuspended in 200 μL of 0.1N NaOH, incubated for 5 min at RT, spun down, and resuspended in 50–70 μL sample buffer (3.5% SDS, 13.8% glycerol, 120mM Tris base, 8mM EDTA, 5% β-mercaptoethanol, Protein Inhibitor, 0.005% Bromophenol blue) incubated in a water bath at 95^◦^C for 7 min. Samples were spun down for min and loaded 0.2–0.25 OD per lane.

Proteins were transferred to a PVDF membrane (Cat # ISEQ00010, Sigma-Aldrich) using Transfer Buffer for 2 h and 30 min at 70V or 30V overnight at 4^◦^C, followed by membrane blocking with 5% Milk Blocking Buffer for 1 h. Anti-GFP primary antibody (1:2000 or 1:1000) from mouse (Cat # 11814460001, Roche) was incubated at 4^◦^C overnight, followed by three 10-min washes with TBS-T. The secondary anti-mouse antibody from sheep (1:5000) (Cat # NA 931V, Cytiva) was incubated for 1 h at room temperature, followed by three 10-min washes with TBS-T. Loading control bands were detected using the same procedure, employing anti-G6PDH antibodies from rabbits (Cat # A9521, Sigma) as primary and anti-rabbit antibodies as secondary (Cat # NA 934V, Cytiva). The signal was detected using SuperSignal PLUS Chemiluminescent Substrate Kit (Cat #34577/34095, Thermo Scientific).

#### Cox4-mCherry mitophagy

Cells expressing cox4-mCherry were inoculated into SD-Leu and grew overnight until the mid-log phase (OD_600_ 0.5–0.8). The next day, cells were collected, spun down, and resuspended in SD-Leu with 2% lactic acid, then diluted into flasks containing SD-Leu with lactic acid at an OD_600_ 0.2. Cells were incubated at 28^◦^C with shaking for 48h. After the 48h incubation, cells were treated with Rapamycin (final concentration 0.2μM) for 4 and 24h. Cells stained with CMAC (Cat #Y7531, Life Technologies) were incubated for 15– 30 min at RT and imaged before and after rapamycin treatment (37).

#### Proteasome inhibition

For proteasome inhibition with MG 132, cells were grown overnight in high proline media (0.17% YNB, 0.1% proline, 2% glucose) up to mid-log phase. Cells were collected, spun down, resuspended in a high proline medium with 0.003% SDS, and incubated at 30^◦^C for 1h. After incubation, cells were treated with 10 mM MG 132 (final concentration 75μM) for 1h.

#### Heat shock induction

Cells were grown overnight in YPD or SD media until mid-log phase at 30^◦^C. After reaching the mid-log phase, cells were moved from 30^◦^C to 39^◦^C for 1h. Cells were imaged before and after the temperature change. Fluorescence intensity per cell was compared from 30^◦^C to 39^◦^C using ImageJ. For ER-phagy cargoes, accumulation of GFP-Snc1-PEM or Snq2-yEGFP in cells was quantified and expressed as “percent cells with GFP signal inside.

#### Heat shock factor 1

Cells expressing activated Heat Shock Factor 1 (Hsf1 R206S) were grown overnight in SD media up to mid-log phase OD_600_ 0.5–1. Once the desired OD was reached, cells were imaged using a Yokogawa Spinning Disk confocal Leica DM8i inverted microscope. Fluorescence intensity per cell was compared between empty vector vs. cells expressing Hsf1 R206S using ImageJ.

#### UPR induction assay

Cells were grown in SD or YPD media to mid-log phase at 28^◦^C, then incubated at 28^◦^C with DMSO or 5 μg/mL tunicamycin for 90min (Cat #T7765, Sigma-Aldrich).

#### β-Galactosidase activity assays

For UPR β-gal assay, cells were transformed with the pNS1254 plasmid and grown with or without 5 μg/mL tunicamycin for 90 min. The level of β-galactosidase in cell lysates was determined by permeabilized cell assay resuspending cells in 700 μL Z buffer, followed by the addition of 50 μL chloroform and 50 μL of 0.1% SDS, and vortex at max speed for 10 s. Samples were preincubated at 30^◦^C for 5min, then started reaction by adding 20 μL of ONP. Samples were incubated at 30^◦^C until the mixture was pale yellow (47). The reaction was stopped by adding 350 μL of Na_2_CO_3_

For the HSR β-gal assay, cells were transformed with pCM64-SSA3 HSE-LacZ alone or together with either pRS423-hsf1-R206S (or pRS423, for a plasmid control), and the permeabilized cell assay was performed by resuspending cells in 700 μL Z buffer, followed by the addition of 50 μL chloroform and 50 μL of 0.1% SDS, and vortex at max speed for 10 s. Samples were preincubated at 30^◦^C for 5min, then started reaction by adding 20 μL of ONP. Samples were incubated at 30^◦^C until the mixture was pale yellow (47). The reaction was stopped by adding 350 μL of Na_2_CO_3_.

For both assays, activity was expressed as β-galactosidase Miller units.

### QUANTIFICATION AND STATISTICAL ANALYSIS

All statistics were performed using either Student’s t-test or one-way ANOVA with Tukey’s post hoc test performed using GraphPad Prism 8.3.0 software. Data are presented as mean ± Standard Deviation (STD) and differences were considered significant at *p* ≤ 0.05.

## Supplementary Material

1

2

3

4

5

6

7

8

9

10

11

12

13

14

15

16

17

## Figures and Tables

**Table:** KEY RESOURCES TABLE

REAGENT or RESOURCE	SOURCE	IDENTIFIER
Antibodies		

Mouse anti-GFP	Roche	11814460001
Rabbit anti-G6PDH	Sigma	A94521
Donkey Amersham ECL Rabbit IgG, HRP-linked whole Ab	Cytiva	NA 934V
Sheep Amersham ECL Mouse IgG, HRP-linked whole Ab	Cytiva	NA 931V

Bacterial and virus strains		

NEB 5-alpha Competent *E*. *coli*	New England BioLabs	C29871

Chemicals, peptides, and recombinant proteins		

Rapamycin	LC Laboratories	R-5000
Trichloroacetic acid (TCA)	Fisher BioReagents	BP555-250
Tunicamycin	Sigma-Aldrich	T7765
β-mercaptoethanol	Bio-Raid	1610710
Protease inhibitor cocktail, complete EDTA-free	Sigma-Aldrich	11873580001
FM4-64	Invitrogen	T3166
CMAC	Life Technologies	Y7531

Experimental models: Organisms/strains		

*Matα his3 Δ200 leu2-3*,*112 ura3-52 gal lys2-801 ade2*	Lipatova et al.^35^	N/A
*Matα his3-Δ200 leu2-3*,*112 ura3-52 ypt1-T40K*	Lipatova et al.^35^	N/A
*Mata leu2 Δ0 ura3 Δ0 his3 Δ1 met15 Δ0*	Brachmann et al.^75^	N/A
NSY825 *atg1Δ*::*KAN*	Lipatova & Segev^6^	N/A
*Mat*α *leu2-3*,*112 ura3-52 his3 Δ200 lys2-801 trp1-1*	Ravid et al.^14^	N/A
*TRP1*::*Deg1-F-Vam12-yEGFP*		
NSY825 *atg17Δ*::*KAN*	Lipatova & Segev^6^	N/A
NSY1646 *SEC61mCherry*::*NAT*	Lipatova & Segev^6^	N/A
NSY825 *atg7*::*G418*	Lipatova et al.^15^	N/A
NSY825 *trs85Δ*::*HYGRO*	Lipatova et al.^15^	N/A
NSY1962 *hac1Δ*::*KAN*	This study	N/A
NSY825 *Rtn1-GFP*::*HYGRO*	This Study	N/A
NSY1646 *Rtn1-GFP*::*HYGRO*	This Study	N/A
NSY825 *Nop1-GFP*::*HYGRO*	This Study	N/A
NSY1646 *Nop1-GFP*::*HYGRO*	This Study	N/A
NSY1962 *doa10Δ*::*KAN*	Lipatova et al.^15^	N/A
NSY1962 *atg9Δ*::*HYGRO*	Lipatova et al.^15^	N/A
NSY2020 *doa10Δ*::*KAN*	Lipatova et al.^15^	N/A
NSY825 *doa10Δ*::*KAN*	Lipatova et al.^15^	N/A
NSY1963 *doa10Δ*::*NAT*	This study	N/A
NSY825 *atg13Δ*::*KAN*	This study	N/A
NSY1962 *atg1Δ*:: *HYGRO*	This study	N/A
NSY2017 *atg1Δ*:: *HYGRO*	This study	N/A
NSY2017 *pep4Δ*:: *HYGRO*	This study	N/A
NSY825 *pep4Δ*:: *HYGRO*	This study	N/A
NSY1894 *pep4Δ*::*HYGRO*	This study	N/A
NSY2017 *vps27Δ*::*NAT*	This study	N/A
NSY825 *vps27Δ*::*NAT*	This study	N/A
NSY1894 v*ps27Δ*:*NAT*	This study	N/A
NSY1962 *atg7Δ*::*NAT*	This study	N/A
NSY2017 *atg7Δ*::*NAT*	This study	N/A
NSY2017 *atg1Δ*::*HYGRO*, *rsp5Δ*:NAT with pRS316-Rsp5	This study	N/A
NSY2017 *atg1Δ*::*HYGRO*, *rsp5Δ*:NAT with pRS316-*Rsp5 L733S*	This study	N/A
NSY2017 *atg1Δ*::*HYGRO*, *ssh4Δ*:NAT	This study	N/A

Recombinant DNA		

pJC104 *4xUPRE1-*crippled *CYC1* promoter*-lacZ*, for UPR analysis, 2μm, *URA3*, Amp^r^	Cox and Water^76^	N/A
pRS425-GFP-Snc1PEM GFP-Snc1-PEM, 2μm, *Leu2*, Amp^r^	Lipatova et al.^35^	N/A
pRS425-Snq2yEGFP *ADH1 promotor-Snq2-yEGFP-CYC1 terminator in pRS425*	Lipatova et al.^15^	N/A
pRS425 2μm, *Leu2*, Amp^r^	Christianson et al.^77^	N/A
pJU676 *pRS416*-Sch9-5XHA, CEN, *Ura3*, Amp^r^	Urban et al.^48^	N/A
*PCM64-SSA3 HSE lacZ SSA3-HSE* *fused to lacZ*, for heat shock analysis, 2μm, *URA3*, Amp^r^	Liu et al.^49^ Liu and Chang^21^	N/A
Yep96 *hsf1-R206S* with its own promoter and terminator, 2μm, *Trp1*, Amp^r^	Sewell et al.^42^ Liu and Chang^21^	N/A
pRS423 2μm, *His3*, Amp^r^	Christianson et al.^77^	N/A
pRS316-*Rsp5-WT Rsp5-WT* with its own promoter and terminator, *Ura3*, Amp^r^	Li et al.^32^	N/A
pRS316-*Rsp5-L733S Rsp5-L733S* with its own promoter and terminator, *Ura3*, Amp^r^	Li et al.^32^	N/A
pHS12-mCherry Encodes mitochondrially targeted preCox4 (22amino acid)-mCherry under ADH1 promoter, *Leu2*, Amp^r^		Addgene Plasmid #25444
PRS423-*Hsf1-R206S hsf1-R206S* with its own promoter and terminator, 2μm, *His3*, Amp^r^	This study	N/A

Software and algorithms		

ImageJ	NIH	https://imagej.net/
GraphPad Prism	GraphPad Software Inc.	https://www.graphpad.com/

## Highlights

- Micro-ER-phagy clears aberrant membrane proteins early under nutritional stress
- Macro-autophagy degrades normal proteins later
- This selective pathway requires a ubiquitin ligase and ESCRT
- Visualization of live engulfment of cargo by the lysosome
