# Supplementary material for: Selective clearance of aberrant membrane proteins by TORC1-mediated micro-ER-phagy
Source: Cell Rep. Author manuscript; Available in PMC 2025 Apr 15. (PMC11999474; doi:10.1016/j.celrep.2025.115282)
Supplement: 1 [file NIHMS2060935-supplement-1.pdf]

Cell Reports, Volume *44*

Supplemental information

Selective clearance of aberrant membrane proteins by TORC1-mediated micro-ER-phagy

Valeriya Gyurkovska, Yaneris M. Alvarado Cartagena, Rakhilya Murtazina, Sarah F. Zhao, Candela Ximenez de Olaso, and Nava Segev

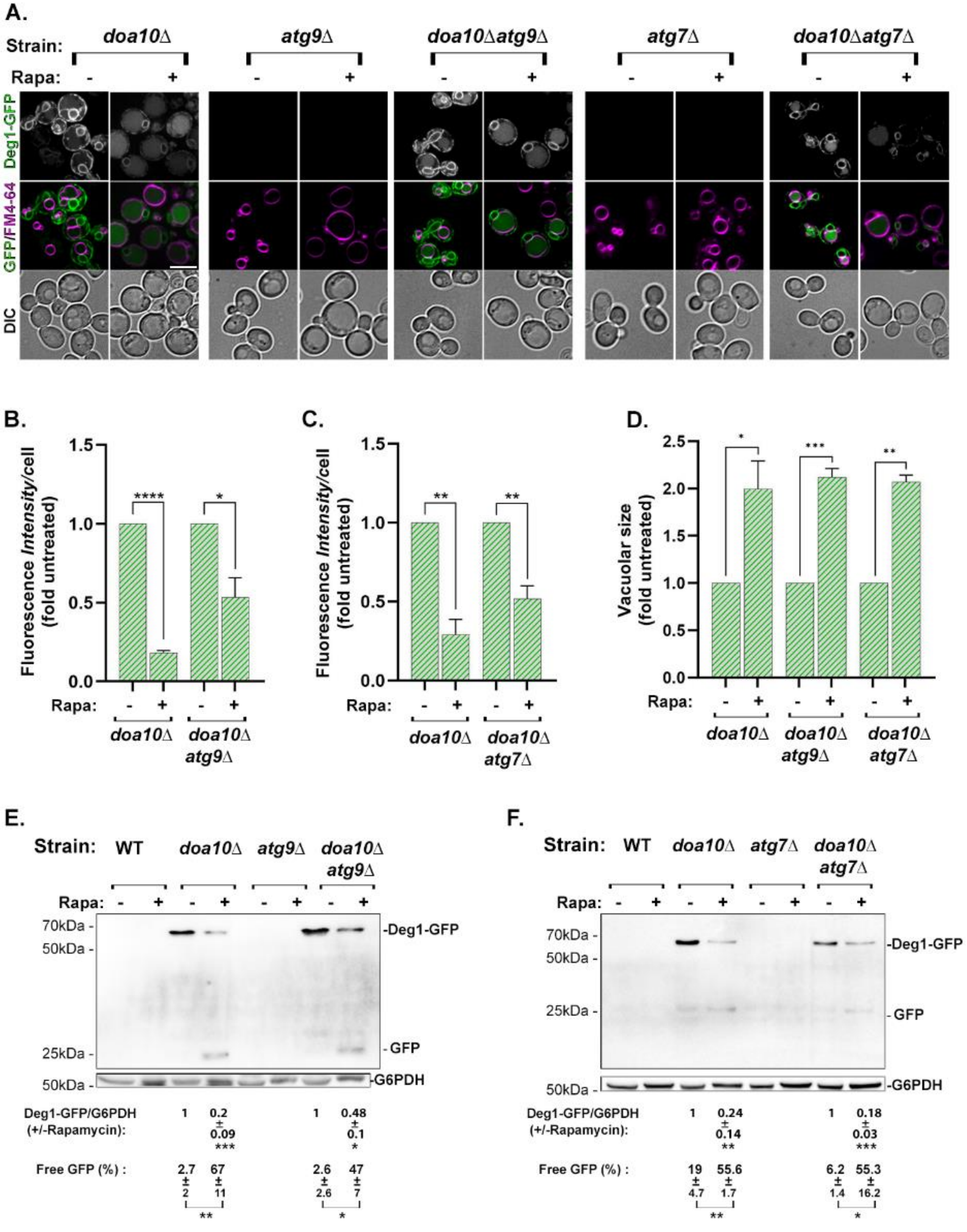

Figure S1: Rapamycin induces Deg1-GFP clearance in an Atg9-and Atg7-independent manner. WT, *doa10*Δ; *atg9*Δ, and *doa10*Δ *atg9*Δ; *atg7*Δ, and *doa10*Δ *atg7*Δ mutant cells expressing Deg1-GFP were treated with rapamycin (200nM, 4 hours) and tested by microscopy (A-D) and immunoblot analyses (E-F)

as described for Figure 1. **A.** Representative images: From top to bottom: Strain, growth condition (-/+ rapamycin), GFP, GFP + FM4-64 merge, DIC. Scale bar, 10 μm**. B-C.** Bar graphs showing quantification of GFP fluorescence intensity per cell (in cells from panel A) as fold change from untreated cells (set to 1) for *doa10*Δ, and *doa10*Δ *atg9*Δ (B), and *doa10*Δ, *doa10*Δ *atg7*Δ (C). **D.** Bar graph showing quantification of vacuole size (in cells from panel A) as fold change from untreated cells (set to 1). After rapamycin treatment the fluorescence intensity significantly decreases while the vacuole size increases in *doa10*Δ, *doa10*Δ *atg9*Δ, and *doa10*Δ *atg7*Δ mutant cells. **E-F.** Immunoblot analyses: Cell lysates (from panel A) were subjected to immunoblot analysis using anti-GFP antibody. Representative blot showing from top to bottom: Strain, treatment (- or + rapamycin), GFP blot, G6PDH blot (loading control), and quantification of Deg1-GFP (corrected by the loading control, and compared between the same culture before and after rapamycin), +/- and significance, quantification of free GFP % of Total GFP in the same lane, +/- and significance. The majority (>75%, >50% and >80%) of the Deg1-GFP that accumulated in *doa10*Δ, *doa10*Δ *atg9*Δ and *doa10*Δ *atg7*Δ mutant cells, respectively, was cleared by rapamycin treatment. Error bars represent mean ± STD; ns, non-significant, *p<0.05, **p<0.01, ***p<0.001, ****p<0.0001. Results in this figure represent three independent experiments.

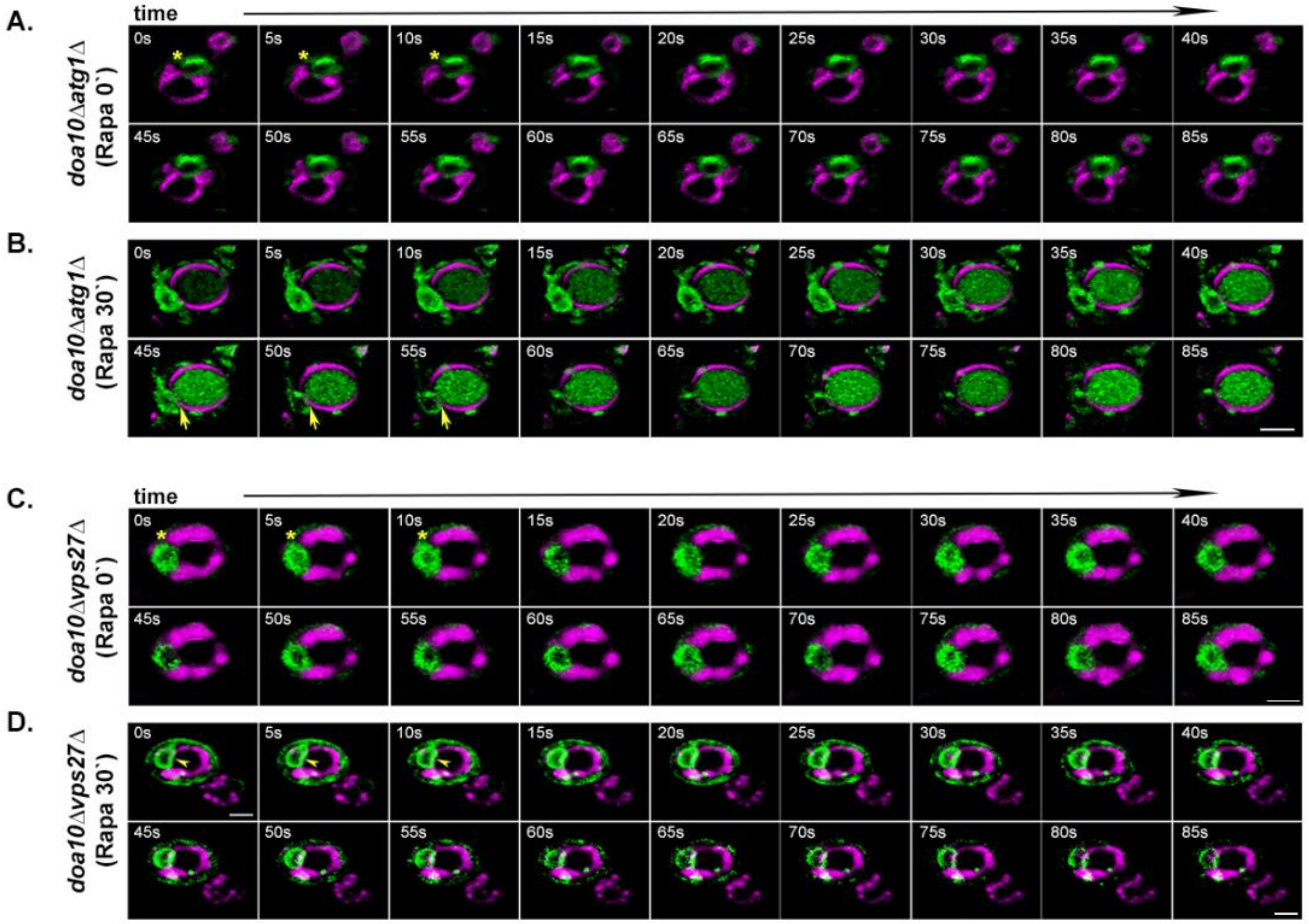

Figure S2: Timelapse fluorescence microscopy of Deg1-Vma12-GFP in *atg1*Δ and *vps27*Δ mutant cells. A-D. shown are still frames from SV 1, 3, 5, and 7, respectively. All cells express Deg1-Vma12-GFP (green), and vacuolar membrane is stained with FM4-64 (magenta). Video acquisition: double lasers, every 5 sec 0.5 μm/section. **A.** *doa10*Δ *atg1*Δ cells, before addition of rapamycin; green does not enter the lysosome; asterisks mark frames used for SV2. **B.** *doa10*Δ *atg1*Δ cells, 30 min after addition of rapamycin; arrows point to sites of GFP entrance to the lysosome in frames used for SV4. **C.** *doa10*Δ *vps27*Δ cells, before addition of rapamycin; green does not enter the lysosome; asterisks mark frames used for SV6. **D.** *doa10*Δ *vps27*Δ cells, 30 min after addition of rapamycin. Colocalization of GFP and FM4-64 is, arrowheads point to sites of GFP-FM4-64 colocalization (seen as white) in frames used for SV8. Results in this figure represent three independent experiments.

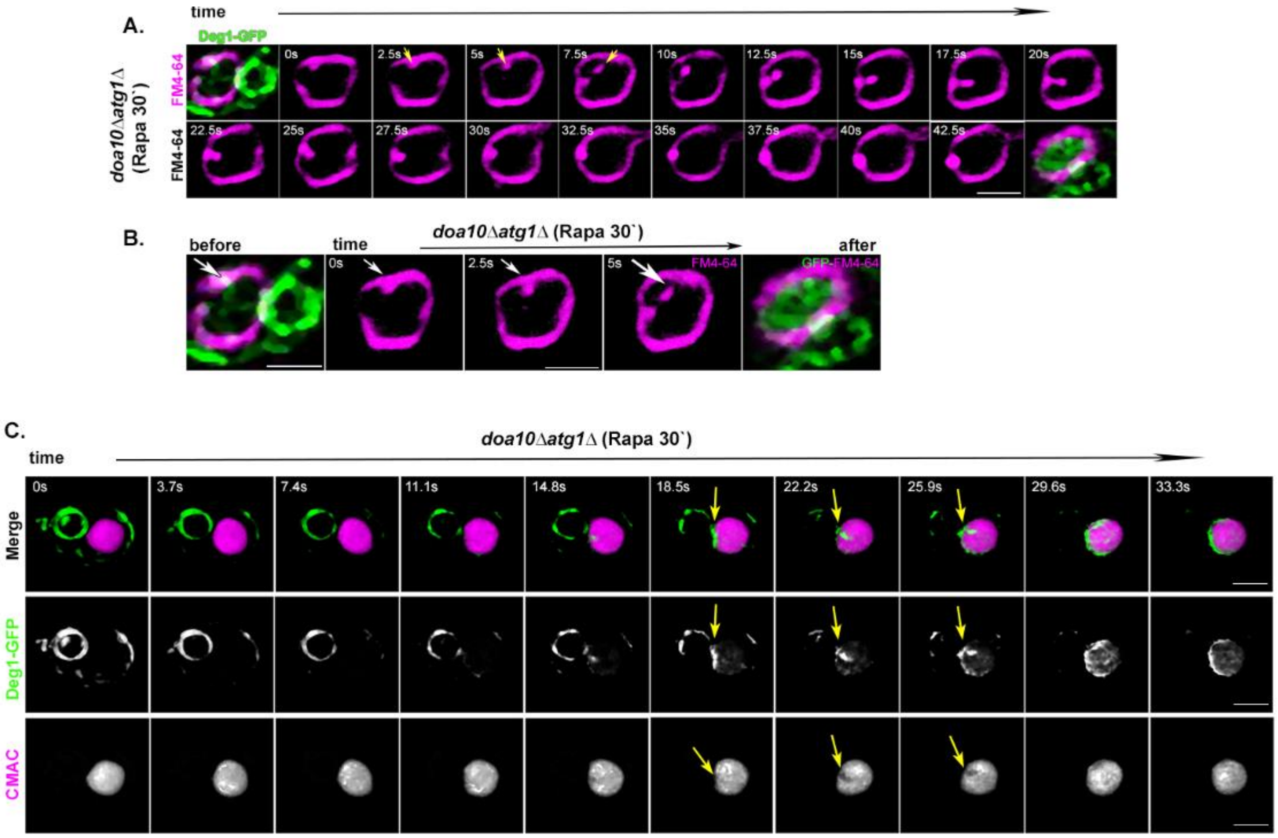

Figure S3: Fast acquisition of timelapse fluorescence microscopy of Deg1-Vma12-GFP in *atg1*∆ mutant cells after addition of rapamycin. Timelapse microscopy of *doa10*∆ *atg1*∆ cells expressing Deg1-Vma12-GFP (green) 30 min after addition of rapamycin. **A-B.** Lysosomal membrane stained with FM4-64 (magenta); video acquisition: single laser, every 2.5 sec 0.25 μm/section. First and last frames in show snap shots of GFP + FM4-64 in the beginning and end of video, respectively. **A.** Timelapse still frames of SV9 are shown; arrows point to lysosomal membrane invagination in frames used for SV10. **B.** Dynamics of the lysosomal membrane invagination; frames from SV10 showing before, during and after vacuolar membrane invagination Arrows point to the invagination site. **C.** Timelapse still frames from SV11: Lysosomal lumen stained with CMAC (magenta). Video acquisition: double lasers, every 3.7 sec 0.25 μm/section taken with a multi-path filter. Arrows point to sites of GFP entrance to the lysosome in frames used for SV12. Scale bar, 2 μm. Results in this figure represent three independent experiments.

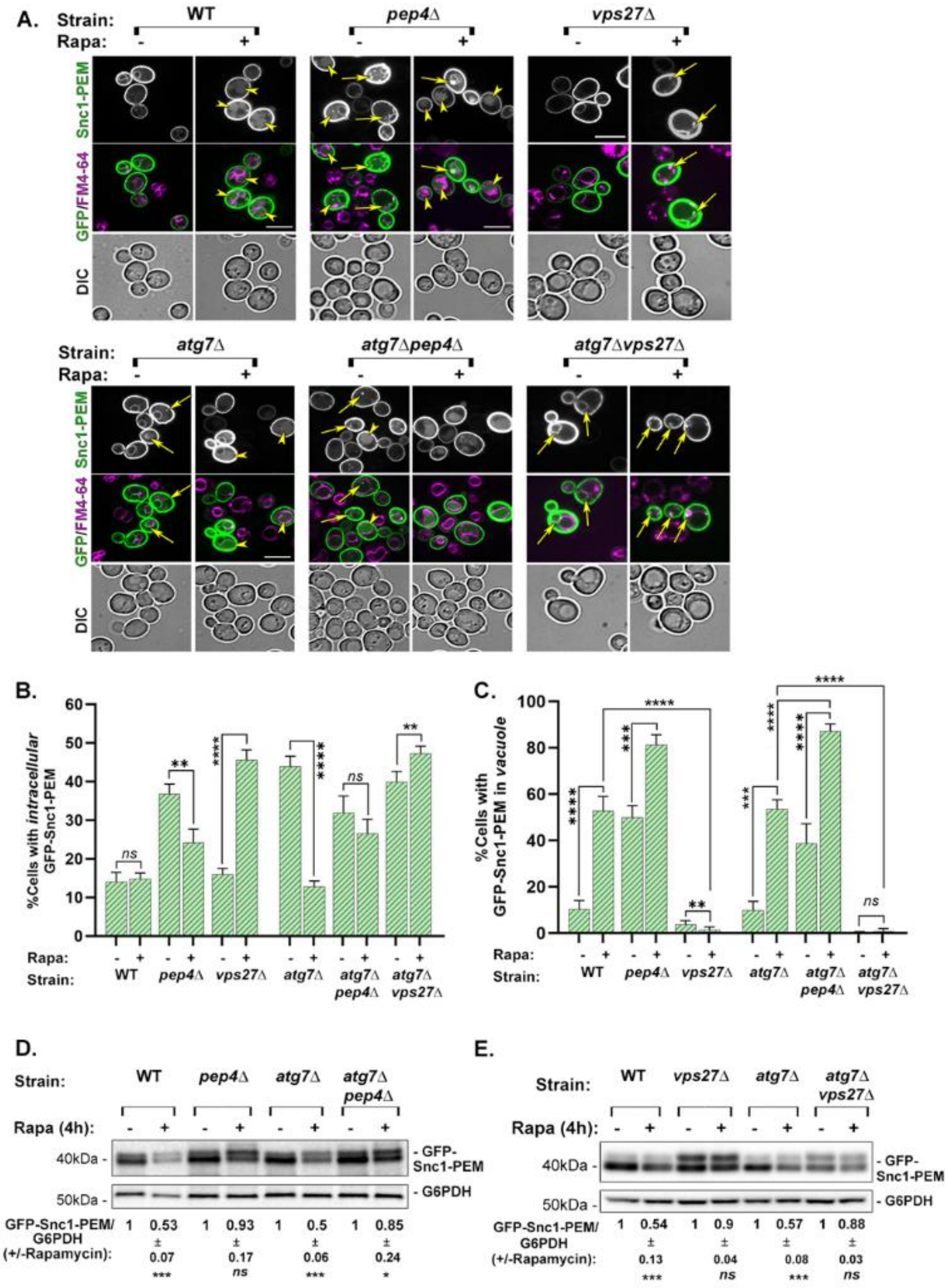

Figure S4: Rapamycin-induced clearance of an ER-phagy cargo occurs in the lysosome/vacuole and depends on ESCRT. The following six strains, WT, *pep4*Δ, *vps27*Δ, *atg7*Δ, *atg7*Δ *pep4*Δ, and *atg7*Δ *vps27*Δ (NSY825, NSY2128, NSY2142, NSY1894, NSY2130, and NSY2143, respectively) were

transformed with a plasmid for expression of GFP-Snc1-PEM. Cells were grown and treated with rapamycin as described in Figure 1 legend and visualized by live-cell confocal microcopy (A-C) and immunoblot analysis (D-E). **A-C.** microscopy: **A.** Representative images: Shown from top to bottom: strain, medium (-/+ rapamycin), GFP-Snc1-PEM, merge of GFP and FM4-64, and DIC. Arrows point to intracellular GFP-Snc1-PEM; and arrowheads to GFP inside the vacuole; scale bar, 10 µm. **B-C.** Quantification of microscopy described in panel A: Bar graphs show percent cells with aberrant intracellular GFP-Snc1-PEM (**B**) and with GFP-Snc1-PEM inside the vacuole (**C**). Whereas intracellular GFP-Snc1-PEM is cleared upon rapamycin treatment from *atg7*∆ mutant cells (71%), it is not cleared in *atg7*∆ *pep4*∆ and *atg7*∆ *vps27*∆ double-mutant cells. Before and after rapamycin treatment, more *atg7*∆ *pep4*∆ double mutant cells accumulate GFP in their vacuole (after, >80%) than *atg7*∆ mutant cells (after, ~50%). In contrast, GFP does not reach the vacuole of *vps27*∆ and atg7∆ *vps27*∆ mutant cells. **D-E.** Immunoblot analysis of lysates made from cells from panel A. Representative blot showing WT, *pep4*∆*, atg7*∆, *and atg7*∆ *pep4*∆, (**D**), and WT, *vps27*∆*, atg7*and *atg7*∆ *vps27*∆ (**E**). From top to bottom: Strain, treatment (-/+ rapamycin), GFP blot, G6PDH blot, quantification of GFP-Snc1-PEM (as described for Figure 1), +/- and significance. MW markers, left; analyzed proteins, right (G6PDH, loading control). Whereas the level of GFP-Snc1-PEM is decreased by ~50% under rapamycin treatment in WT and *atg7*∆ mutant cells, it does not decrease in *pep4*∆*, and atg7*∆ *pep4*∆ (D), nor in *vps27*∆ and *atg7*∆ *vps27*∆ (E). Error bars represent mean ± STD; ns, non-significant, *p<0.05, **p<0.01, ***p<0.001, ****p<0.0001. Results in this figure represent four independent experiments.

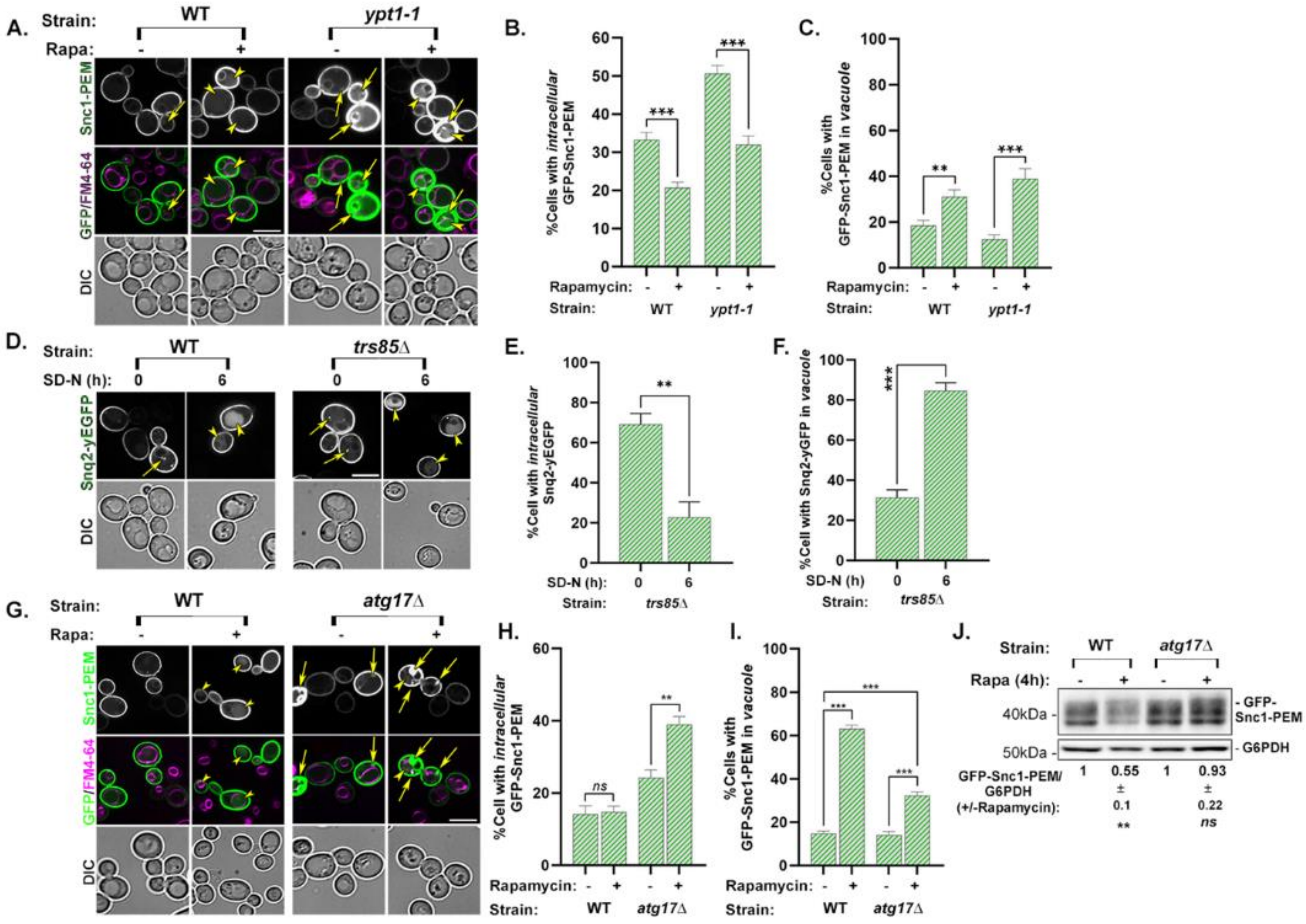

Figure S5: Nutritional stress effect on macro-ER-phagy cargos that accumulate in *ypt1-1 trs85*Δ, and *atg17*Δ mutant cells. A-C. Clearance of GFP-Snc1-PEM during rapamycin treatment in *ypt1-1* mutant cells. The following two strains, WT and *ypt1-1* (NSY128, NSY55, respectively) were transformed, grown and visualized as described for Figure 6A. **A.** Representative images. Bar graphs of cells from panel A, show percent cells with aberrant intracellular GFP-Snc1-PEM (**B**) and with GFP-Snc1-PEM inside the vacuole (**C**). Rapamycin treatment results in clearance of intracellular GFP-Snc1-PEM and its delivery to the vacuole in *ypt1-1* mutant cells. **D-F.** Clearance of Snq2-yEGFP during nitrogen starvation in *trs85*Δ mutant cells. WT and *trs85*Δ (NSY825, NSY1922, respectively) were transformed with a plasmid to overexpress Snq2-yEGFP. Cells were grown in regular media (SD, Time 0) or shifted to medium without nitrogen (SD-N) for 6 hours. **D.** Representative images: Shown from top to bottom: strain, SD-N (0 or 6 hours), Snq2-yEGFP, and DIC. Bar graphs of cells from panel D, show percent cells with aberrant intracellular Snq2-yEGFP (**E**) and with GFP inside the vacuole (**F**). Nitrogen starvation results in clearance of intracellular Snq2-yEGFP (>70%) and its delivery to the vacuole (>80%) in *trs85*Δ mutant cells. In panels A and D: Arrows point to intracellular GFP-Snc1-PEM and Snq2-yEGFP; and arrowheads to GFP inside the vacuole; scale bar, 10 µm. **G-J.** Effect of rapamycin on GFP-Snc1-PEM in *atg17*Δ mutant cells. WT and *atg17*Δ mutant cells (NSY825 and NSY1610, respectively) were transformed with a plasmid for overexpression of GFP-Snc1-PEM, grown, treated and analyzed by microscopy (G-J) and immunoblot (I) analyses. Microscopy images (representative, **G**) were quantified: percent cells with aberrant intracellular GFP-Snc1-PEM (**H**) and with GFP inside the vacuole (**I**). Rapamycin treatment does

not induce clearance of GFP-Snc1-PEM, which accumulates further in *atg17*Δ mutant cells (~50% more) and its delivery to the vacuole is lower than in other core Atg-depleted mutant cells (~30%, compared to ~60% in *atg1*Δ and *atg7*Δ mutant cells. **J.** Immunoblot representative (as in Figure 5). The level of GFP-Snc1-PEM is not decreased in *atg17*Δ mutant cells. Error bars represent mean ± STD; ns, non-significant, *p<0.05, **p<0.01, ***p<0.001, ****p<0.0001. Results in this figure represent three independent experiments.

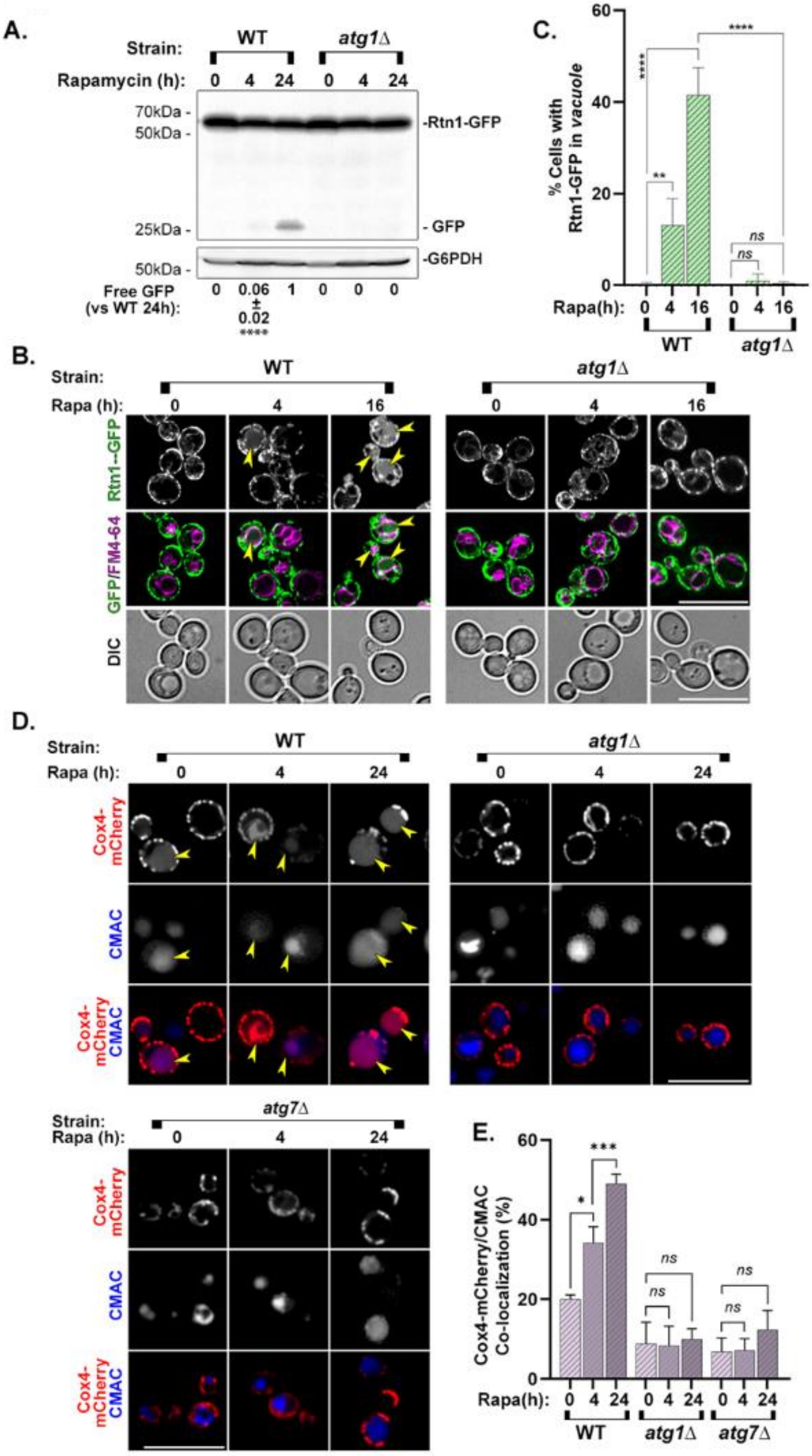

Figure S6: Normal ER-membrane and excess mitochondrial proteins are not cleared by nutritional stress induced micro-autophagy. A-C. Degradation of endogenously expressed Rtn1-GFP, an ER

membrane protein, during rapamycin treatment is dependent on Atg1**.** **A.** Upon rapamycin treatment, Rtn1-GFP is degraded to release free GFP in WT, but not *agt1*Δ mutant cells. WT and *atg1*Δ mutant cells (NSY1986 and NSY1988, respectively) expressing Rtn1-GFP from their endogenous locus were grown in regular media (0 hours) or with rapamycin (200 nM for 4 or 24 hours). Immunoblots (representative blots shown) of cell lysates: From top to bottom: strain, growth conditions, GFP blot showing Deg1-GFP and Free GFP, G6PDH blot (loading control), and Free GFP (fold over WT + rapamycin 24 hours; mean, ± STD). **B-C.** Upon rapamycin treatment, Rtn1-GFP reaches the lysosome in WT, but not *atg1*Δ mutant cells. **B.** WT and *atg1*Δ mutant cells from panel A grown in regular media (0 hours) or with rapamycin (200 nM for 4 or 16 hours). The lysosomal membrane was stained by FM4-64 (for 30 minutes; see Methods) and the cells were visualized by live-cell fluorescence microscopy. Representative images are shown. From top to bottom: Strain (WT, left; atg1Δ, right), growth condition (0, 4, 16 hours with rapamycin), GFP, GFP + FM4-64 merge, DIC. Scale bar, 10 μm**.** **C.** Bar graph showing quantification of percent cells (from panel B) with GFP in the lysosome. GFP can be seen in lysosomes of WT cells, but not of *atg1*Δ mutant cells. **D-E.** Delivery of the mitochondrial marker Cox4-mCherry during rapamycin treatment is dependent on Atg1 and Atg7. WT, *atg1*Δ and *atg7*Δ mutant cells were transformed with a plasmid (pHS12-mCherry) for expression of the mitochondrial marker Cox4-mCherry under the ADH1 promoter. Cells were treated with rapamycin (200 nM for 0, 4 or 24 hours), the lysosomal lumen was stained with CMAC, and the cells were visualized by live-cell fluorescence microscopy. Representative images are shown: From top to bottom: Strain, growth condition (0, 4, 24 hours with rapamycin), Cox4-mCherry, CMAC, and Cox4-mCherry + CMAC merge. Scale bar, 10 μm**.** **E.** Bar graph showing quantification of percent cells (from panel D) with Cox4-mCherry in the lysosome (co-localization of mCherry and CMAC). After rapamycin treatment Cox4-mCherry can be seen in lysosomes of WT cells, but not of *atg1*Δ or *atg7*Δ mutant cells. Error bars represent mean ± STD; ns, non-significant, *p<0.05, **p<0.01, ***p<0.001, ****p<0.0001. Results in this figure represent three independent experiments.

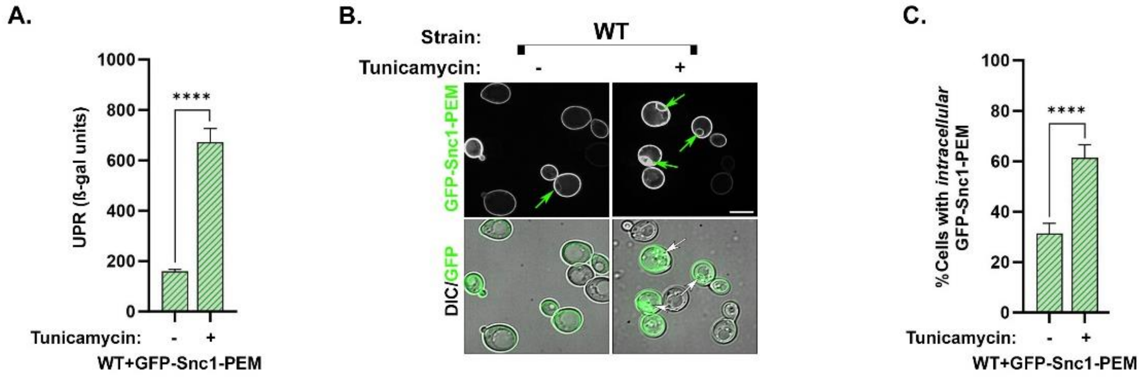

Figure S7: The effect of ER stress on ER-QC cargos. A-C. ER stress exacerbates the accumulation of an ER-phagy cargo. WT cells (NSY825) were transformed with two plasmids, one for overexpression of GFP-Snc1-PEM (pNS1407), and the other for expression of the LacZ gene under a UPR-inducible promoter (pNS1254). Cells were grown treated with Tunicamycin and UPR was determined as described in Fig S9A (A) and by live-cell microscopy (B-C). **A.** UPR was induced in cells treated with Tunicamycin by >3 fold. **B-C.** Tunicamycin-induced ER stress results in increase in the percent of WT cells that accumulate intracellular GFP-Snc1-PEM**. B.** Microscopy: Representative images of untreated (left) and tunicamycin treated cells (right) visualized by live cell microscopy (GFP, top; GFP/DIC merge, bottom). Arrows point to intracellular GFP-Snc1-PEM; scale bar, 10 µm. **C.** Bar graph showing quantification of the percent of cells with intracellular GFP-Snc1-PEM from panel B, which increases by ~2-fold after treatment with Tunicamycin. Error bars represent mean ± STD, ****$p<0.0001$. Results in this figure represent three independent experiments.

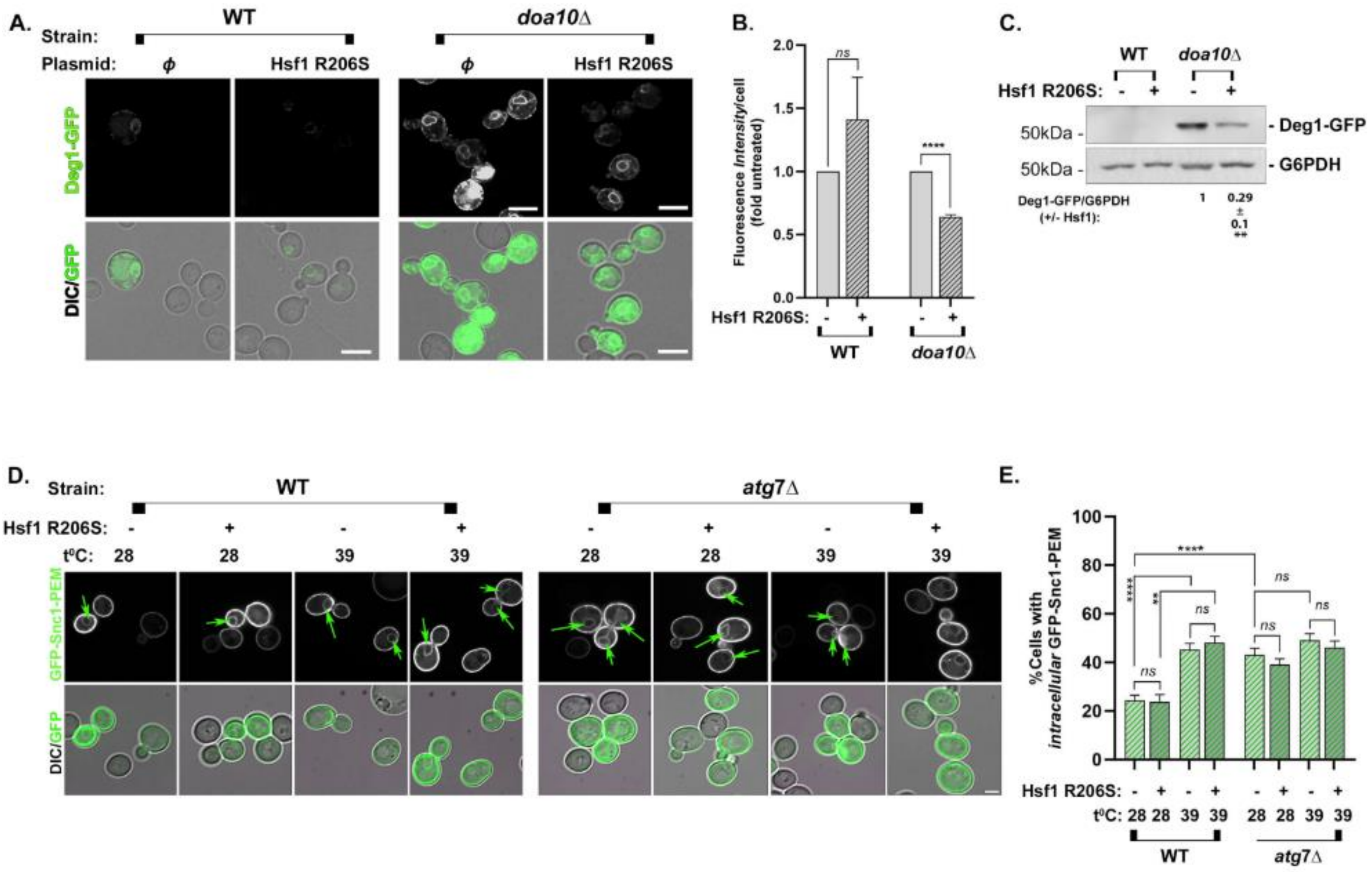

Figure S8. HSR affects clearance of ERAD, but not ER-macro-phagy, cargo. A-C. Effect of HSR on Deg1-GFP level using microscopy (A-B) and immunoblot (C) analyses. **A.** Microscopy: WT (left) and *doa10*Δ mutant (right) cells expressing Deg1-Vma12-GFP (NSY1962 and NSY2017, respectively) were transformed with an empty plasmid (q, pNS1709) or a plasmid expressing activated Hsf1, Hsf1-R206S (pNS1711). Cells were grown at 28°C and visualized by live-cell microscopy. Shown from top to bottom: Strain, plasmid, GFP Fluorescence, GFP+DIC merge (for visualizing cells); scale bar, 5 µm. Very little fluorescence is seen in WT cells expressing Deg1-Vma12-GFP, and the protein accumulates in *doa10*Δ mutant cells. **B.** Bar graph shows Deg1-GFP fluorescence intensity/cell from cells described in panel D. In *doa10*Δ mutant cells growing at 28°C expressing Hsf1-R206S (black pattern) the level of Deg1-GFP fluorescence is significantly lower (36%) when compared to cells transformed with empty plasmid (solid bars). The fluorescence level in WT cells is low and expression of Hsf1-R206S does not elicit a significant difference. **C.** Immunoblot analysis: Lysates from WT and *doa10*Δ mutant cells expressing Deg1-Vma12-GFP, without or with expression of Hsf1-R206S (from panel A), were tested by immunoblot analysis using anti-GFP antibodies to detect GFP-Snc1-PEM (top), and anti-G6PDH antibodies (loading control). Shown representative blot: on left to right: WT cells not expressing and expressing Hsf1-R206S, and *doa10*Δ mutant cells not expressing and expressing Hsf1-R206S (MW markers shown on the left). Shown top to bottom: Strain, plasmid (empty or Hsf1-R206S), blot, quantification of Deg1-GFP (corrected by the loading control, and compared between cells expressing or not expressing Hsf), +/- and significance. Activated Hsf1 effect: Deg1-GFP protein level is significantly lower in *doa10*Δ mutant cells expressing Hsf1-R206S (~70%) when compared to cells transformed with empty plasmid (Deg1-GFP protein level in WT cells is below the detection level). **D-E.** Heat, but not HSR, causes accumulation of macro-ER-phagy cargo. WT and *atg7*Δ mutant cells (NSY825, NSY1894, respectively) were transformed with two plasmids: one for overexpression of GFP-Snc1-PEM (pNS1407, or pRS425 as an empty plasmid control) and the other for expression of Hsf1-R206S (pNS1711, or pRS423 as an empty plasmid control). Cells were

grown at 28°C or shifted to 39°C for 1 hour and tested by live-cell microscopy. **D.** Representative images: Shown from top to bottom: Strain, Hsf1 R206S, growth temperature, GFP Fluorescence, GFP+DIC merge (for visualizing cells). Most of the GFP-Snc1-PEM is on the PM, but some accumulates inside cells. Arrows point to intracellular GFP-Snc1-PEM; scale bar, 10 µm. **E.** Bar graph showing quantification of the microscopy experiment (D). While shifting WT cells to 39°C for 1 hour results in a significant increase (~2-fold) in the percent of cells with internal GFP-Snc1-PEM (as seen in Figure 6), expression of Hsf1-R206S does not have this effect at 28°C (ns). The percent of *atg7*Δ mutant cells with internal GFP-Snc1-PEM is higher than in WT cells and is not increased upon heat treatment or expression of Hsf1-R206S. In this figure: Error bars represent mean ± STD; ns, non-significant, *p<0.05, **p<0.01, ***p<0.001, ****p<0.0001. Results in this figure represent three independent experiments.

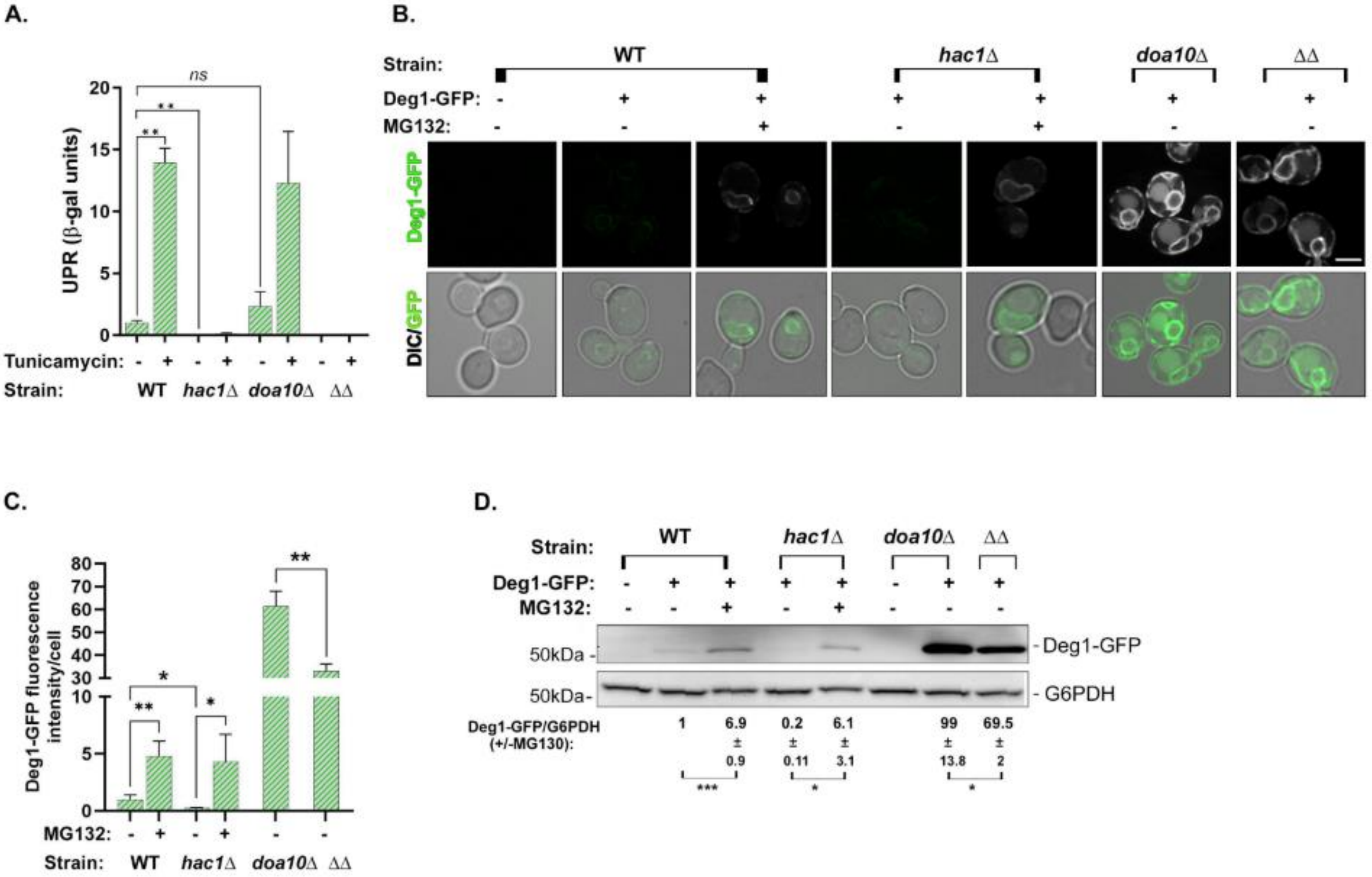

Figure S9. UPR does not play a role in clearance of an ERAD-C substrate during normal growth. A. UPR induction in cells expressing Deg1-Vma12-GFP. WT (NSY1962), *doa10*Δ *(*NSY2017*)*, *hac1*Δ (NSY1963) and *doa10*Δ *hac1*Δ (NSY2073) mutant cells expressing Deg1-Vma12-GFP were transformed with a plasmid for expression of the LacZ gene under a UPR-inducible promoter (pNS1254). Cells were grown under normal conditions without or with 5 µg/ml tunicamycin (90 min). UPR was determined as β-galactosidase units in cell lysates. Bar graph (all have green pattern for Deg1-GFP expression) shows UPR as fold of β-gal in wild-type cells. Tunicamycin was used as a control to show that UPR can be induced in WT and *doa10*Δ mutant cells. First, UPR is not induced because of accumulation of Deg1-Vma12-GFP in doa10Δ mutant cells (compare Deg1-GFP accumulation in panel B, and UPR level here, ns, between WT and *doa10*Δ). Second, induction of UPR by ER stress is abolished in cells deleted for *HAC1* (*hac1*Δ and *doa10*Δ *hac1*Δ (used below*)*. Error bars represent mean +/- STD (ns, the difference between WT and *doa10*Δ is not-significant). **B-D.** Deg1-Vma12-GFP clearance in wild-type cells, or accumulation in *doa10*Δ mutant cells, is not diminished by UPR abolition. WT (NSY1962), *doa10*Δ *(*NSY2017*)*, *hac1*Δ (NSY1963) and *doa10*Δ *hac1*Δ (NSY2073) mutant cells expressing Deg1-Vma12-GFP were grown under normal conditions. The proteasomal inhibitor MG132 (75 µM, 1 hour, in high proline medium, [75]) was used to show that Deg1-Vma12-GFP is expressed in WT and *hac1*Δ mutant cells (where it is cleared efficiently by ERAD-C). Cells that do not express Deg1-GFP (NSY825) are used as a negative control. The cells were visualized by live-cell fluorescence microscopy (B-C) and lysates were subjected to immuno-blot analysis using anti-GFP antibodies (D-E). **B-C.** Microscopy: (B) Representative images: Shown from top to bottom: Strain, Deg1-GFP, MG132, GFP Fluorescence, GFP+DIC merge (for visualizing cells). (C) Bar graph shows quantification of average fluorescence (mean +/-STD) from cells described in panel B, statistical significance; scale bar, 5 µm. **D.** Immuno-blot analysis: Representative immunoblot: Shown from top to bottom: Strain, GFP blot, G6PDH blot (loading control), fold Deg1-GFP

(over wild type), mean ± STD; ns, non-significant, *p<0.05, **p<0.01, ***p<0.001, ****p<0.0001. Microscopy and immunoblot analyses show that whereas Deg1-Vma12-GFP accumulates in *doa10*Δ and *doa10*Δ *hac1*Δ mutant cells, it does not accumulate in WT and *hac1*Δ mutant cells; some accumulation is seen in WT and *hac1*Δ mutant cells treated with MG132. Importantly, the level of Deg1-Vma12-GFP does not increase upon deletion of *HAC1*, showing the UPR is not required for clearance of this ERAD-C cargo. Results in this figure represent three independent experiments.

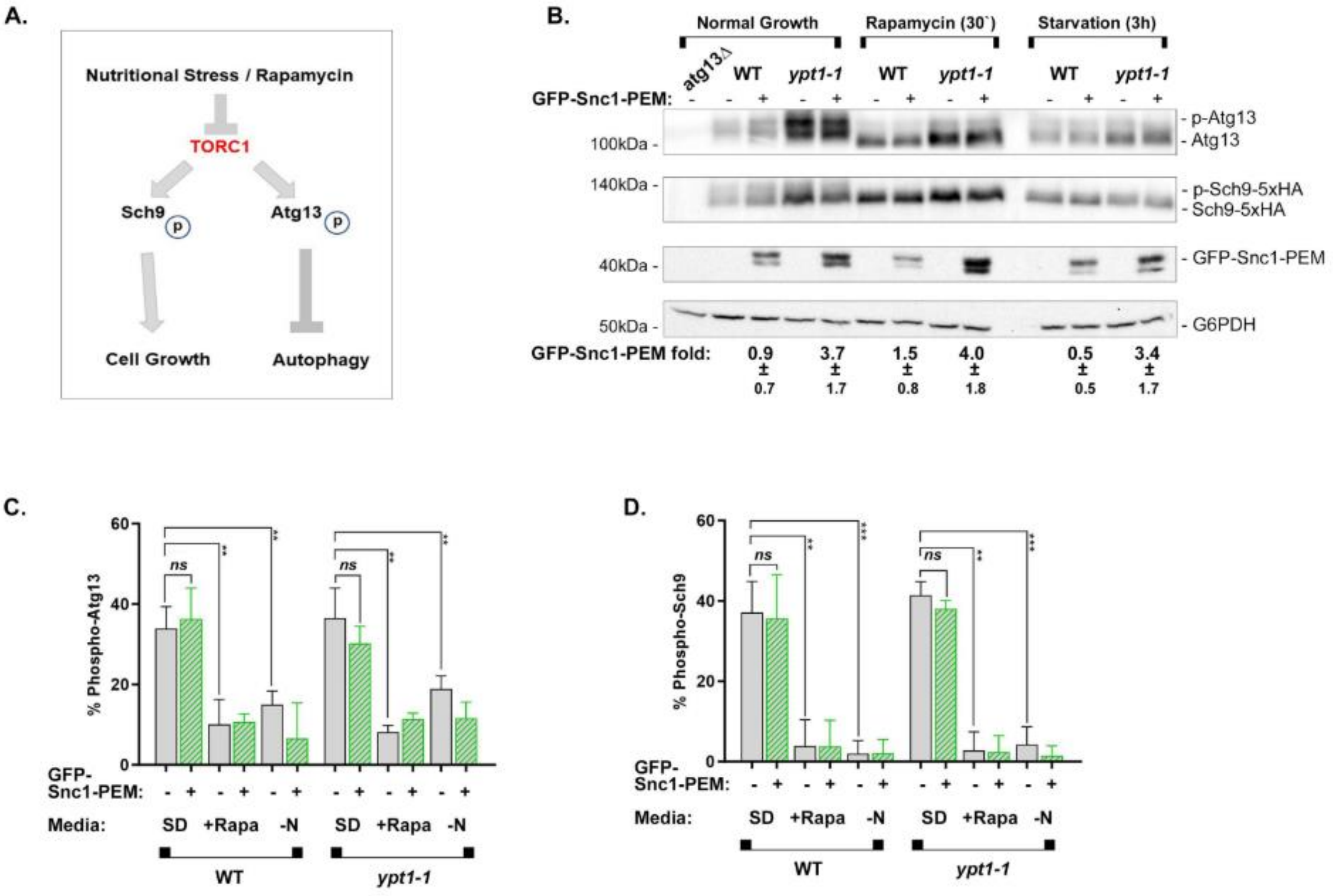

Figure S10. Macro-ER-phagy during normal growth is independent of TORC1 signaling: Atg13 and Sch9 phosphorylation. A. Diagram showing the two downstream effectors of TORC1 kinase used here: First, phosphorylation of Atg13 by TORC1 inhibits autophagy (right); Second, Sch9 is phosphorylated directly by TORC1 to promote cell growth (left). As a result, under normal growth conditions, cell growth is promoted, and autophagy is inhibited. In contrast, nutritional stress or addition of rapamycin inhibits TORC1 activity resulting in Atg13 dephosphorylation and autophagy induction, and Sch9 dephosphorylation and growth inhibition. **B.** Atg13 and Sch9 phosphorylation is not changed during clearance (in wild-type, WT, cells) or accumulation (in *ypt1-1* mutant cells) of the macro-ER-phagy cargo GFP-Snc1-PEM. WT (NSY825) and *ypt1-1* (NSY55) mutant cells were transformed with two plasmids: one for expression of Sch9-HA and the second for overexpression of GFP-Snc1-PEM (pRS425 as an empty plasmid control). Cells were grown under normal conditions (SD medium, to select for the plasmids). For positive controls to show TORC1 inhibition, cells were treated with rapamycin (200 nM for 30 minutes), or starved for nitrogen (for 3 hours). Cell pellets were lysed and used for an immunoblot analysis. Representative blot: from top to bottom: anti-Atg13 antibodies (p-Atg13 and Atg13), anti-HA antibodies to detect Sch9-HA (p-Sch9 and Sch9), anti-GFP antibodies to detect GFP-Snc1-PEM, and anti-G6PDH antibodies (loading control). Shown on the left, *atg13Δ* (NSY2080) mutant cells as a negative control for cells that do not express either Atg13 or Sch9-HA. In each growth condition, the four lanes show WT cells without and with GFP-Snc1-PEM, and *ypt1-1* without and with GFP-Snc1-PEM (MW markers shown on the left). Fold accumulation of GFP-Snc1-PEM is shown at the bottom; *ypt1-1* mutant cells accumulate about 4-fold over the WT under normal growth conditions or short nutritional stress (30 minutes rapamycin or 3h nitrogen starvation; note: this nutritional stress is sufficient for induction of the signaling pathway, but much shorter than that used below for testing clearance of GFP-Snc1-PEM). **C.** Bar graph showing p-Atg13 as percent of total Atg13. **D.** Bar graph showing p-Sch9 as percent of total

Sch9-HA. There is no difference in the phosphorylation level of Atg13 or Sch9 between WT or *ypt1-1* mutant cells overexpressing and not overexpressing GFP-Snc1-PEM. As a control, significant difference in dephosphorylation of Atg13 (C) and Sch9 (D) is seen in cells (WT and *ypt1-1*) grown with rapamycin or nitrogen starvation when compared to cells grown under normal growth conditions (SD). Bar graphs: + cargo (GFP-Snc1-PEM), green pattern; error bars represent mean ± STD; ns, non-significant, *p<0.05, **p<0.01, ***p<0.001, ****p<0.0001. Results in this figure represent three independent experiments.

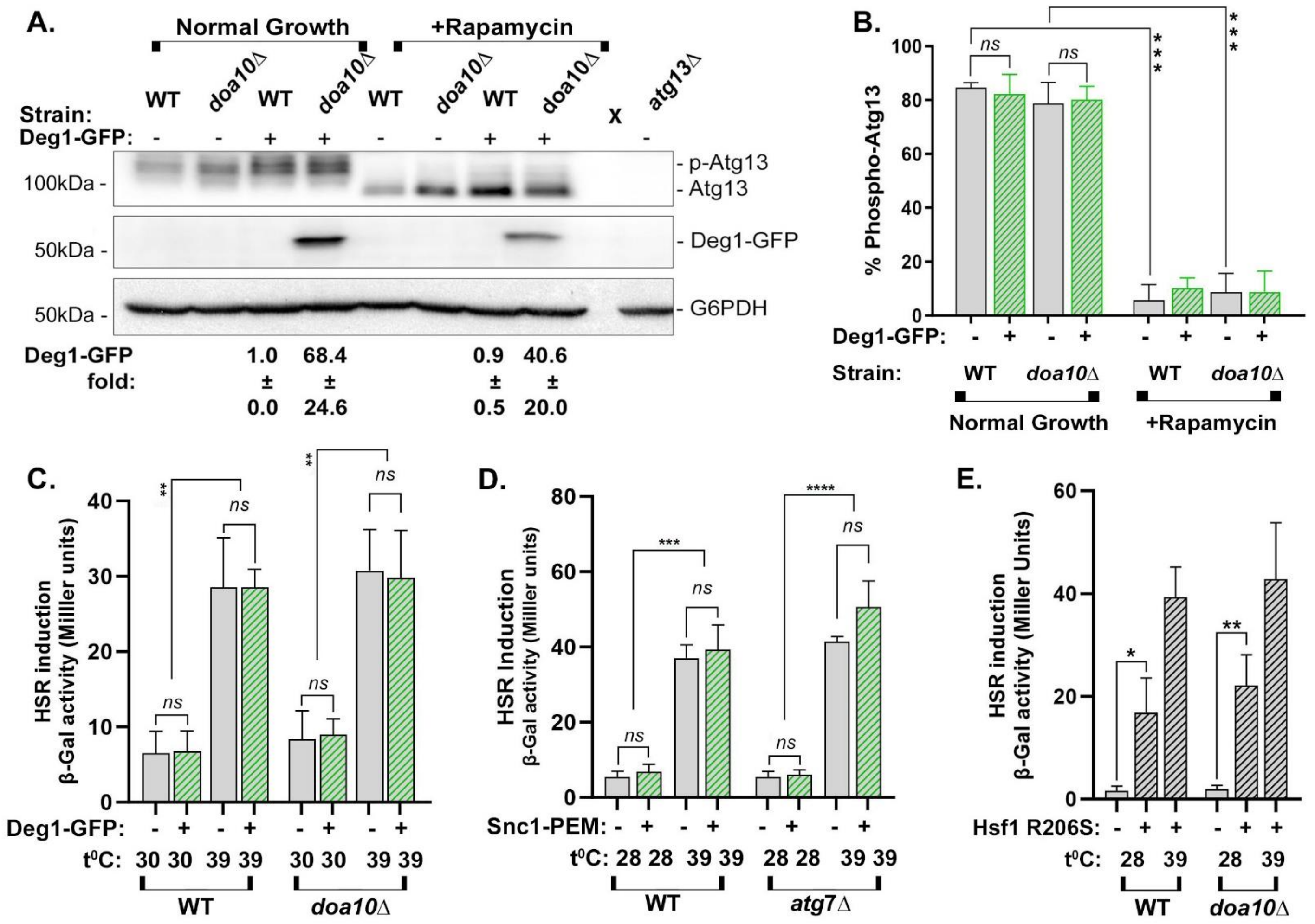

Figure S11. Clearance of aberrant membrane proteins and TORC1 and HSR signaling. A-B. TORC1 signaling is not induced during clearance of an ERAD-C substrate during normal growth. WT (NSY1962) and *doa10*Δ (NSY2017) mutant cells expressing Deg1-Vma12-GFP from Figure 1 were grown in normal medium (SD) or with rapamycin (SD+rapa, 200 nM for 30 minutes). WT (NSY825) and *doa10*Δ (NSY2025) not expressing Deg1-Vma12-GFP were used as negative controls. **A.** Cell lysates were tested by immunoblot analysis (representative blot) using anti-Atg13 antibodies to determine Atg13 phosphorylation status. From top to bottom: growth conditions, strains, Atg13 blot (p-Atg13 and Atg13), GFP blot to determine Deg1-GFP level, G6PDH blot (loading control), fold Deg1-GFP level over WT (mean ± STD). Left to right: MW markers, normal medium, + rapamycin, *atg13*Δ (NSY2080; negative control) (X marks empty lane). Importantly, expression of Deg1-Vma12-GFP does not change the phosphorylation level of Atg13 in WT cells, which clear Deg1-GFP, or in *doa10*Δ mutant cells, which accumulate it but can still clear some of it by macro-ER-phagy (see text), under normal growth conditions. Dephosphorylated Atg13 is seen only in cells grown with rapamycin. **B.** Bar graph showing percent of phosphorylated Atg13 in cells from panel A. **C-E.** HSR. **C.** HSR is not induced by expression of Deg1-Vma12-GFP in WT cells or its accumulation *in doa10*Δ mutant cells at 30°C. WT and *doa10*Δ mutant cells not expressing (NSY825 and NSY2025, respectively) or expressing Deg1-Vma12-GFP (NSY1962 and NSY2017, respectively) were transformed with plasmid for expression of the LacZ gene under an HSE-inducible promoter (pNS1683). Cells were grown at 30°C or shifted to 39°C for 1 hour. HSR was determined as β-galactosidase units. Bar graph shows HSR as β-gal activity (Miller units) in WT (left) and *doa10*Δ mutant cells (right) not expressing Deg1-Vma12-GFP (solid bars) or expressing Deg1-Vma12-GFP (green stripped bars) grown at 30°C or 39°C (1h). There is no significant difference between HSR

induction in WT or *doa10*Δ mutant cells expressing or not expressing Deg1-Vma12-GFP. HSR is significantly induced only when cells were exposed to 39°C (as a control). **D.** Effect of macro-ER-phagy cargo on HSR. WT and *atg7*Δ mutant cells (NSY825, NSY1894, respectively) were transformed with two plasmids: one for overexpression of GFP-Snc1-PEM (pNS1407, or pRS425 as an empty plasmid control), and the other for expression of the LacZ gene under a HSE-inducible promoter (pNS1683) (See panel B). Cells were grown at 28°C or shifted to 39°C for 1 hour and HSR was determined as described in panel C. Bar graph shows: WT (left) and *atg7*Δ mutant cells (right) not expressing GFP-Snc1-PEM (solid bars) or expressing GFP-Snc1-PEM (green pattern) grown at 28°C or 39°C (1h). HSR is not induced by expression of GFP-Snc1-PEM in WT or *atg7*Δ mutant cells at 28°C. HSR is significantly induced only when cells were exposed to 39°C (as a control). **E.** Expression of activated heat shock factor-1, Hsf1-R206S, induces HSR. WT or *doa10*Δ mutant cells expressing Hsf1-R206S grown at 28°C (39°C is shown as a positive control for induction of HRS response by heat). HSR was determined as β-galactosidase units in cell lysates. Bar graph shows HSR as described for panel-C. Bar graph shows: WT (left) and *doa10*Δ mutant cells (right) all expressing Deg1-Vma12-GFP grown at 28°C or 39°C (1h). HSR is induced in WT or *doa10*Δ mutant cells expressing Hsf1-R206S (black pattern) growing at 28°C, about half of the HSR induced by heat (39°C, 1h). In B-D, + cargo, green pattern; Error bars represent mean ± STD; ns, non-significant, *$p<0.05$, **$p<0.01$, ***$p<0.001$, ****$p<0.0001$. Results in this figure represent three independent experiments.

Table S1. Speed of micro-ER-phagy events

Method*	Lasers	Interval (sec)	Total time (sec)	Events	Total cells	%	Rate of events (events/sec/cell) **	Speed of micro-ER-phagy (sec)
1	Double	5	90	79	210	30%	$7.37x10^{-4}$ (+/- $2.4x10^{-4}$)	**12.9** +/-3.4
2	Single	2.5	55	132	300	31%	$11.96x10^{-4}$ (+/- $3.8x10^{-4}$)	**7.3** +/-1.7
3	Double	3.7	58	42	130	32%	$18.7x10^{-4}$ (+/- $4.0x10^{-4}$)	**8.8** +/-2.0

* Method number, refer to text
**p-values for rate: non-significant, 0.25 method 1 vs 2, 0.063 method 2 vs 3, 0.14 method 1 vs 3
